# Endophenotype trait domains for advancing gene discovery in autism spectrum disorder

**DOI:** 10.1186/s11689-023-09511-y

**Published:** 2023-11-22

**Authors:** Matthew W. Mosconi, Cassandra J. Stevens, Kathryn E. Unruh, Robin Shafer, Jed T. Elison

**Affiliations:** 1 Schiefelbusch Institute for Life Span Studies and Kansas Center for Autism Research and Training (K-CART), University of Kansas, Lawrence, KS USA; 2 Clinical Child Psychology Program, University of Kansas, Lawrence, KS USA; 3 Institute of Child Development, University of Minnesota, Minneapolis, MN USA; 4 Department of Pediatrics, University of Minnesota, Minneapolis, MN USA

## Abstract

Autism spectrum disorder (ASD) is associated with a diverse range of etiological processes, including both genetic and non-genetic causes. For a plurality of individuals with ASD, it is likely that the primary causes involve multiple common inherited variants that individually account for only small levels of variation in phenotypic outcomes. This genetic landscape creates a major challenge for detecting small but important pathogenic effects associated with ASD. To address similar challenges, separate fields of medicine have identified endophenotypes, or discrete, quantitative traits that reflect genetic likelihood for a particular clinical condition and leveraged the study of these traits to map polygenic mechanisms and advance more personalized therapeutic strategies for complex diseases. Endophenotypes represent a distinct class of biomarkers useful for understanding genetic contributions to psychiatric and developmental disorders because they are embedded within the causal chain between genotype and clinical phenotype, and they are more proximal to the action of the gene(s) than behavioral traits. Despite their demonstrated power for guiding new understanding of complex genetic structures of clinical conditions, few endophenotypes associated with ASD have been identified and integrated into family genetic studies. In this review, we argue that advancing knowledge of the complex pathogenic processes that contribute to ASD can be accelerated by refocusing attention toward identifying endophenotypic traits reflective of inherited mechanisms. This pivot requires renewed emphasis on study designs with measurement of familial co-variation including infant sibling studies, family trio and quad designs, and analysis of monozygotic and dizygotic twin concordance for select trait dimensions. We also emphasize that clarification of endophenotypic traits necessarily will involve integration of transdiagnostic approaches as candidate traits likely reflect liability for multiple clinical conditions and often are agnostic to diagnostic boundaries. Multiple candidate endophenotypes associated with ASD likelihood are described, and we propose a new focus on the analysis of “endophenotype trait domains” (ETDs), or traits measured across multiple levels (e.g., molecular, cellular, neural system, neuropsychological) along the causal pathway from genes to behavior. To inform our central argument for research efforts toward ETD discovery, we first provide a brief review of the concept of endophenotypes and their application to psychiatry. Next, we highlight key criteria for determining the value of candidate endophenotypes, including unique considerations for the study of ASD. Descriptions of different study designs for assessing endophenotypes in ASD research then are offered, including analysis of how select patterns of results may help prioritize candidate traits in future research. We also present multiple candidate ETDs that collectively cover a breadth of clinical phenomena associated with ASD, including social, language/communication, cognitive control, and sensorimotor processes. These ETDs are described because they represent promising targets for gene discovery related to clinical autistic traits, and they serve as models for analysis of separate candidate domains that may inform understanding of inherited etiological processes associated with ASD as well as overlapping neurodevelopmental disorders.

## Background

Autism spectrum disorder (ASD) is both clinically and etiologically diverse. Rare inherited and de novo pathogenic variants each have been repeatedly implicated and account for up to 20% of cases [1, 2]. For a plurality of individuals with ASD, however, it is believed that the primary causes include gene-gene and gene-environment interactions that involve multiple common inherited variants and non-linear complex genetics [3, 4]. Consistent with this hypothesis, > 1000 different genes show associations with ASD, and the majority of variants implicated each confer only small effects [5]. These findings suggest that many different pathogenic processes contribute to ASD, and that additive or multiplicative genetic effects play prominent roles in the development of autism. This polygenic landscape also suggests significant etiological heterogeneity among autistic individuals, indicating studies of large numbers of individuals will be necessary to help parse the many distinct causal pathways involved. Large, multi-site research networks and data sharing consortia have been leveraged to establish a growing number of candidate genes and greater understanding of their downstream molecular consequences in relation to ASD [6–9]. Despite these efforts, diagnostic yield from genetic testing in ASD remains low and etiological processes for most autistic individuals remain unexplained [10, 11]. Additional approaches are needed to increase resolution for detecting small but significant genetic effects and advance more individualized identification and therapeutic strategies.

One approach to gene discovery that has proven valuable in separate fields of medicine is the identification of condition-related biological traits that can be used to decompose complex clinical phenotypes into less genetically complex trait structures. Endophenotypes, or discrete, quantitative traits that reflect genetic likelihood for a particular clinical condition, have been used to identify polygenic mechanisms of complex conditions and advance more personalized therapeutic strategies for heart disease [12, 13], obesity [14, 15], diabetes [16], and osteoporosis [17, 18]. Endophenotypes represent a distinct class of biomarkers useful for understanding genetic contributions to clinical entities because they are (A) embedded within the causal chain between genotype and clinical phenotype, (B) closer to the action of the gene(s) than the constellation of clinical phenotypes that define a diagnosis, and (C) quantitative and therefore capable of showing greater sensitivity to additive causal processes than categorical outcomes, such as affectation status. Despite their demonstrated power for guiding new understanding of complex genetic structures of clinical conditions, few endophenotypes associated with ASD have been identified and integrated into family genetic studies.

In this paper, we argue that understanding the complex pathogenic processes that contribute to ASD can be accelerated by refocusing attention toward identifying endophenotypic traits reflective of inherited mechanisms. This pivot requires renewed emphasis on study designs integrating measurement of familial co-variation including infant sibling studies, family trio and quad designs, and analysis of monozygotic and dizygotic twin concordance for select trait dimensions. Given that most, if not all behavioral traits associated with ASD also are implicated in separate behaviorally defined disorders (e.g., repetitive sensorimotor mannerisms are common in intellectual and developmental disability; difficulties with modulating eye contact during interaction also have been demonstrated in multiple anxiety disorders) and show wide variation in neurotypical development, it is likely that endophenotypic traits associated with ASD will cut across diagnostic boundaries, and transdiagnostic designs will be critical.

**Fig. 1:**
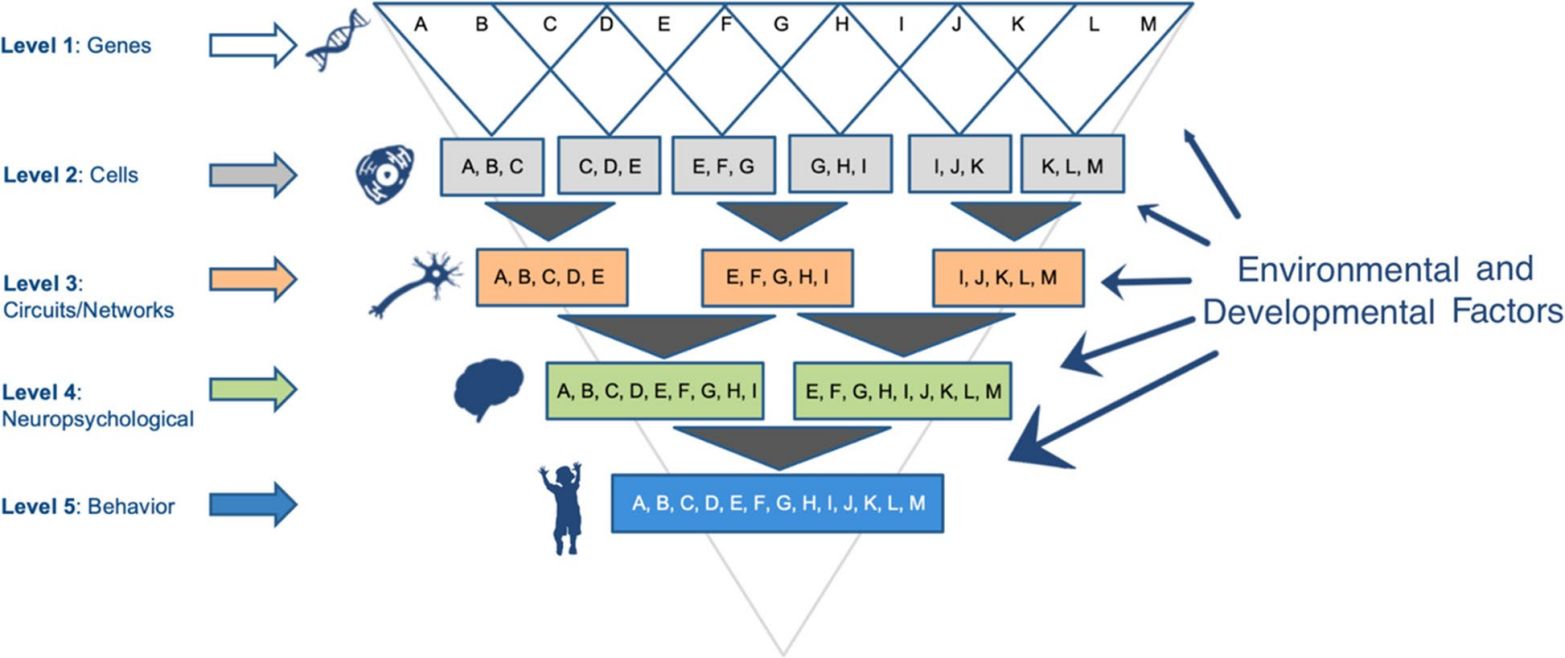
Levels of analysis for mapping etiological pathways associated with behavioral and clinical traits. This schematic shows different layers or functional units of analysis that can be evaluated to clarify linkages between genotype and clinical phenotype. Endophenotypic traits closer to the level of genotype are expected to be more closely associated with inherited variation given their relatively simpler genetic architecture compared to behavioral traits, as evidenced above by the reduced complexity (i.e., number of genes or letters) at the higher levels (e.g., cells, circuits/networks). Multiple levels of analysis are depicted, though separate intermediate levels are not included for ease of presentation (e.g., proteomic). Based on this model, analysis of traits closer to genotypes will provide greater sensitivity to inherited variations than assessments of behavior or complex clusters of clinical symptoms. Analysis of traits across multiple levels, or establishment of endophenotypic trait domains (ETDs), also is proposed to offer unique opportunities for understanding etiological pathways contributing to discrete traits associated with ASD. Important environmental and developmental factors also are proposed to modify trait associations across levels and over time

Multiple candidate endophenotypes associated with ASD likelihood are described herein, though the intent is not to provide a comprehensive review (for more systematic reviews, see [19, 20]), but instead to focus on promising targets for accelerating progress in understanding etiological processes associated with traits involved in ASD. Toward this goal, we propose a new focus on the analysis of “endophenotype trait domains” (ETDs), or traits measured across multiple levels along the causal pathway from genes to behavior. To elucidate ETDs, “dense-phenotyping” approaches will be integral to establishing within-individual associations between traits across molecular, cellular, circuit, system, and behavioral levels, as has been done in separate fields of medicine and areas of psychiatry (see Fig. 1). For example, the Bipolar and Schizophrenia Network for Intermediate Phenotypes (BSNIP) is an on-going, multi-site, transdiagnostic study focused on identifying quantitative traits, measured across multiple levels of analysis, that are associated with psychosis and co-segregate in patients and their first-degree relatives. Studying > 1000 patients affected by psychiatric disorders associated with psychosis (e.g., schizophrenia, bipolar disorder), this network has leveraged broad phenotyping, including measurement of sensory, motor, behavioral, and psychiatric traits, and dense phenotyping strategies, utilizing multiple measures of target domains including genetic, immunological, electrophysiological, oculomotor, functional and structural MR imaging, cognitive, and clinical assays, to derive data-driven “biotypes”, or biologically distinct subgroups of patients [21]. Initial principal components analysis and k-means clustering were used to first derive latent factors across behavioral, cognitive, and brain levels, and then to identify clusters of more biologically homogeneous subtypes. Subsequent work has shown that these biotypes differ on separate external validation characteristics, including brain morphometry, and that affected and unaffected family members co-segregate into similar biotypes implicating high levels of familiality. Similar approaches have been advanced in separate fields of medicine, but no known efforts such as these have been conducted to understand biotypes based on ETDs for neurodevelopmental conditions, including autism.

The concept of ETDs is drawn from the NIMH Research Domain Criteria (RDoC) that focuses on the analysis of domains of behavior from molecular to self-report levels [22], but extends this idea to focus on traits that represent key intermediary processes between genetic causes and select behavioral or clinical dimensions. Here, we emphasize the importance of quantitative trait outcomes rather than categorical diagnoses. This emphasis is critical in the context of considerable evidence that ASD, similar to other psychiatric disorders defined behaviorally by DSM, represent heterogeneous clusters of individuals showing both shared and distinct quantitative deviations from typical or normative profiles of functioning rather than any discovered entity grounded in nature or biology [23]. By mapping endophenotypes across multiple levels and across the full range of neurodiversity, ETDs may provide important insights into pathways of inherited traits that contribute to clinical vulnerabilities and maladaptive developmental functioning. Illustrative examples of how this concept can be applied to understand mechanistic pathways associated with ASD are provided.

To inform our central argument for research efforts toward ETD discovery, we first provide a brief review of the concept of endophenotypes and their application to psychiatry. Next, we highlight key criteria for determining the value of candidate endophenotypes, including unique considerations for the study of ASD and neurodevelopmental disorders more broadly. Descriptions of different study designs for assessing endophenotypes in ASD research then are offered, including analysis of how select patterns of results may help prioritize candidate traits in future research. We then present multiple candidate ETDs that collectively cover a breadth of clinical phenomena associated with ASD, including social, language/communication, cognitive control, and sensorimotor processes. These ETDs are described because they represent promising targets for gene discovery related to clinical traits associated with ASD, and they serve as models for analysis of separate candidate domains that may inform understanding of inherited etiological processes contributing to ASD or associated neurodevelopmental traits.

## Endophenotypes in psychiatry and their application to ASD

The endophenotype concept was first introduced in insect biology to describe “microscopic and internal traits” that contrast “exophenotypes”, or behaviors that are directly observable [24]. Gottesman and Shields [25] initially brought the concept to psychiatry asserting that endophenotypes could provide greater sensitivity to inherited risk factors for psychopathology because they are “a measurable trait that is not observable by the unaided eye… and that lies more proximal to the underlying genetics of a disorder than the clinical phenotype” [26]. Endophenotypes thus represent a unique class of biomarkers that is necessarily influenced by the genetic factors that confer susceptibility to a particular clinical condition [27, 28]. Consistent with this broader definition, Gould and Gottesman [28] proposed key criteria for evaluating the viability of biomarkers as endophenotypic traits. Briefly, the authors indicated that a candidate endophenotype should be (1) associated with condition-specific traits as demonstrated by its presence in affected individuals and covariation with primary trait dimensions in the population, (2) associated with clinical severity within affected individuals, (3) familial, meaning it shows decreasing levels of severity from affected individuals to unaffected family members to unaffected population controls, (4) heritable, and (5) reliably measured and reproducible [26, 29]. Original definitions of endophenotypes also indicated that they should be “state-independent and detectable regardless of whether an individual is acutely ill or in remission”, though recent amendments to these criteria have been proposed to include traits that are observed “prior to the manifestation of features that define a given condition/disorder” that “may or may not persist through development” [30]. Evidence suggests that the familial traits most predictive of autistic outcomes may in fact be deviations previously considered associated features (e.g., motor control features, attention impairments), rather than (or in addition to) core social-communication or repetitive behavior features that appear to show less predictive value early in ontogeny [31]. Moreover, Mous and colleagues [32] found that attention-deficit/hyperactivity disorder (ADHD) and motor coordination traits in both siblings with family history of autism with ASD (FH^+^) and without ASD (FH^−^), strongly predicted ASD trait severity of their autistic sibling and categorical recurrence of ASD within participants’ families (i.e., accounting for ~ 50% of variance). These results suggest that genetic variation underlying non-specific neurodevelopmental traits may represent background ASD susceptibilities that are inherited and non-specific and may confer additive genetic risk alongside variants conferring specific ASD liability (e.g., BAP and subclinical ASD features in parents). These non-specific traits may be detectable earlier than other ASD-related behaviors and serve as critical early targets for intervention. Together, these results suggest that expanding our endophenotype definition to account for a broader range of traits and their developmental variance is crucial.

Several factors have impeded progress in identifying endophenotypic traits useful for gene discovery in ASD. First, studies aimed at understanding the genetics of ASD are inherently constrained by their reliance on categorical definitions that do not have a strong grounding in biology. The diagnostic standards developed by DSM task forces and the gold-standard assessment tools used to inform ASD classifications each primarily were intended to guide reliable, differential diagnoses based on behavioral observation. Historical beliefs that behaviorally defined categories would structure research that could eventually illuminate distinct pathogenic processes for select disorders have not been borne out, likely reflecting the high level of clinical overlap across diagnostic categories and the profound heterogeneity within categories. This realization was explicitly acknowledged by the American Psychological Association prior to publication of DSM-5 when they indicated “historical aspiration of achieving diagnostic homogeneity by progressive subtyping within disorder categories no longer is sensible” ([33], p. 12). It is not surprising then that separate behaviorally defined neurodevelopmental disorder categories (e.g., ASD, ADHD, and obsessive-compulsive disorder, or OCD) each show considerable etiological heterogeneity and overlapping genetic backgrounds [34–36]. Case-control designs that rely on categorical determinations for participant selection also do not capture the full range of trait variation useful for understanding additive genetic effects as demonstrated by population studies showing that defining and associated characteristics of ASD each are normally distributed within the non-autistic population and overlap considerably among autistic and non-autistic individuals [37, 38]. These data indicate transdiagnostic or population-based designs consistent with broader efforts in psychiatry (e.g., the Research Domain Criteria structure of NIMH; Hierarchical Taxonomy of Psychopathology consortium) should be emphasized in the pursuit of identifying familial genetic factors contributing to traits associated with ASD [39].

Translation of the endophenotype concept to ASD also is made difficult by the developmental variance seen in both clinical and biological traits across the life span. Clinical signs of ASD emerge within the first years of life (or perhaps earlier) and evolve in non-linear ways, suggesting endophenotypic traits may differ *quantitatively* as a function of the age or developmental level at which individuals are studied. In support of this hypothesis, multiple studies have shown that some behavioral differences seen in young siblings of autistic individuals relative to non-autistic peers are not predictive of eventual affectation status [40–48]. These findings implicate developmental compensations that mask trait expression at later ages among unaffected first-degree relatives [49]. Further, evidence of “normalization” of key behavioral deficits in later childhood or adulthood among autistic individuals and their unaffected relatives [50–53] suggests that mapping growth trajectories of endophenotypic traits will be critical for establishing trait markers sensitive to gene variation and changes in expression over time.

Additional support for a developmental perspective comes from studies showing characteristics reflecting ASD likelihood in infancy are not direct phenocopies of the defining features measured in children or adults implicating *qualitative* differences in trait expression. For example, recent studies of sibling concordance have indicated that trait dimensions often considered co-occurring conditions may present as the earliest indicators of ASD for some individuals (e.g., motor coordination and behavioral control impairments [32]). These data highlight the importance of analyzing developmental traits beyond core ASD features using a “broad” phenotyping strategy in which diverse traits and their neurodevelopmental and molecular substrates are analyzed. This strategy also demands multivariate analytic approaches in which interactions between different trait domains can be examined. Co-occurring specifiers introduced in DSM 5 (e.g., accompanying intellectual or language impairment) offer a useful set of traits that should be considered as they each individually may represent a top-down starting point for identifying biological processes that collectively contribute to neurodevelopmental disorders including ASD (e.g., accompanying language or intellectual impairment). The question of why these associated traits may co-segregate with core autistic traits in some but not other individuals may be best answered by clarifying the etiologic pathways that underpin discrete traits rather than the more complex (and variable) constellation of clinical behaviors.

Sex differences in both etiological processes and clinical outcomes for autistic individuals also are important considerations in the study of autism genetics. ASD disproportionately impacts males relative to females, and there is considerable evidence that males and females with ASD differ clinically [54, 55], at the level of structural and functional brain development [56–58], and genetically [54, 59]. Infant sibling studies have shown that at least some early indicators of ASD hold predictive power for males but not for females [60], suggesting that candidate traits useful for mapping individual pathophysiologies may differ as a function of sex. In contrast, Burrows et al. [61] recently utilized a data-driven approach to derive behavioral factors and map their early childhood trajectories among infant siblings. Results identified similar sex ratios in infants identified in a “high concern” cluster based on both social-communication and restricted behavior dimensions. Similarly, findings form the Baby Siblings Research Consortium suggest that out of ~1800 toddlers, sex differences in cognitive performance and repetitive behaviors are observed across siblings with a family history of autism (FH) and children with no family history of autism (nFH), suggesting that some early emerging sex differences in cognitive and behavioral development do not appear to be ASD-specific but instead reflect sex-dependent variation in developmental processes that may or may not be altered in ASD [62]. These findings highlight the critical need to adjust for sex-specific biases across trait development for establishing new endophenotypes predictive of autistic trait expression. Inclusion of autistic females in ASD research has been increasingly emphasized in recent years, though this subpopulation remains understudied and still constitutes only a small minority of individuals included in ASD research. There is a strong need for systematic comparisons of trait variation in males and females in the search for ETDs.

## Factors affecting the sensitivity of candidate endophenotypes to genotypes of interest

The genetic landscape of many heritable diseases is defined by complex, non-linear, and polygenic architectures that will not map cleanly onto models developed to predict categorical outcomes (e.g., affectation status) that include highly complex clinical pictures and a diverse range of individuals. While monogenic syndromes show tight genotype-phenotype relationships, complex conditions such as ASD involve high levels of polygenicity, environmental influences, and stochastic events that collectively contribute to diverse behavioral and developmental variation. Defining models that are sensitive to the complex polygenic processes associated with ASD will require identification of dimensional traits that co-vary with additive and non-linear likelihood elements across the affected and unaffected population [63]. By prioritizing dimensional traits sensitive to the full range of expression of candidate genes, power can be maximized for detecting important effects of low penetrant genes that may be accounting for important variation in autistic traits.

The extent to which resolution for identifying pathogenic mechanisms is increased depends on multiple features of candidate traits, including their proximity to the action of the gene. Endophenotypic traits constitute “bridges” linking molecular, cellular, and system-level mechanisms and clinical dimensions. Their associations with genetic variation will be stronger if their relative location on these “bridges” is closer to genetic origins. In principle, endophenotypic traits closer to molecular processes that scaffold brain development will show greater power for informing mechanistic models than more downstream traits that vary as a function of epigenetic processes, environmental factors, and stochastic influences. For example, while the most common endophenotypes studied in psychiatry tend to come from cognitive psychology and take a behavior first, “top down” approach to ETD discovery, cognitive traits often have highly complex genetic architectures themselves, suggesting that their added value relative to measurement of behavior may be limited. It also has been argued that some candidate endophenotypes, including electrophysiological traits, also have shown little power for advancing gene discovery in psychiatric conditions because they have such complex genetic architectures themselves, and thus their study will require very large samples (i.e., tens of thousands) [64]. In contrast, assays of molecular structure or function including transcript- or blood-based traits may offer increased power as they likely will be more directly influenced by the genotype of interest. Mapping ETDs beginning with more molecular traits, or using a “bottom-up” approach, may provide important traction for understanding simpler genetic structures related to discrete trait outcomes. The relationships between molecular traits and the signs and symptoms of ASD often are unclear; however, suggesting that integration of endophenotypes across multiple units of analysis (e.g., transcript, brain function, cognitive processing) will be integral to mapping causal pathways.

Endophenotypic traits also will provide greater power for elucidating genotypes of interest if they are highly translatable across species and model systems. Analyses of preclinical model systems allow for more direct interrogation of cellular and molecular processes and can therefore facilitate more detailed descriptions of the pathways linking genetic substrates and behavioral traits. This consideration is especially important in studies of ASD given that defining symptoms are complex and not easily translatable across primate and more primitive species. For example, translation of the social behavioral difficulties experienced by autistic individuals to model systems is difficult given the complexity of these behaviors and limited conservation of social brain network functions across species. Backwards translation of traits that may be more ontogenetically primitive, including sensory and motor processes, as well as traits measured similarly across species, such as brain structural and functional connectivity, may provide critical insights into the genetic, molecular, and cellular bases of autistic traits. This hypothesis has been supported by studies of “endophenotype ranking values (ERV)” applied to traits associated with separate psychiatric diagnoses [27, 65]. The ERV provides a quantitative ranking system based on estimates of standardized genetic covariance of candidate traits with clinical outcomes. ERV studies have indicated that endophenotypes closer to gene action and translatable across species may show the greatest potential to guide a more mechanistic understanding of complex behavioral dimensions [27, 66].

## Study designs and considerations for identifying endophenotypes

Endophenotypic traits are distinct from other biomarker classes because they represent inherited, additive effects related to development of select traits or disease processes. Based on this premise, candidate traits should show high levels of familiality, meaning they both co-segregate within families and track with clinical traits across affected and unaffected family members. To assess the familiality of candidate traits, multiple different family study designs may be leveraged. Integration of findings across these separate designs is important given that each approach is characterized by unique sets of strengths and limitations.

Family studies represent a broad class of methodologies aimed at determining the extent to which discrete traits differ between unaffected relatives and population controls and co-vary across related family members. The underlying assumption of family study designs aimed at identifying candidate endophenotypes is that traits with strong patterns of inheritance should be more similar across first-degree relatives compared to distant relatives and unrelated population controls. According to Gottesman and Gould’s [28] criteria, strong candidate endophenotypes are evidenced by profiles in which the greatest deviations are seen in individuals with a particular condition relative to population controls, but family members show intermediate levels of deviation from controls in the same direction as seen for affected individuals. Both family trio (biological mother, biological father, and autistic offspring) and quad studies (biological parents, affected, and unaffected siblings) will be important for identifying endophenotypes associated with ASD, and initial family studies already have documented cognitive [67], behavioral [68], and brain differences [69] that covary across affected individuals and their biological parents.

Infant sibling designs represent an important family study approach useful for clarifying endophenotypic traits associated with early development in ASD. Based on the high heritability of ASD and findings that ASD recurrence among siblings (13–20% [70, 71]) is considerably higher than base rates in the general population (1–2% [72]), analysis of infant siblings of previously diagnosed children provides important power for identifying both diagnostic predictors and traits associated with ASD that track in family members. Beginning with Bryson et al.’s [73] initial report documenting social and attentional differences in 12-, but not 6-month-old infants with familial history of ASD who later were diagnosed with ASD, infant sibling designs have identified multiple early emerging markers that can be identified years before the age at which children typically are diagnosed [73, 74]. These studies also have identified candidate traits that track in affected (FH^+^) *and* unaffected (FH^−^) siblings, suggesting that they may reflect inherited genetic influences associated with ASD. Traits that are deviant in both FH^+^ and FH^−^ infant siblings relative to same-age nFH population control infants reflect an important class of familial biomarkers that can guide new knowledge of inherited genetic substructures related to liability for autistic traits. Findings that distinguish FH siblings and nFH peers, regardless of diagnostic status, may reflect familial genetics while stepwise patterns of effects (e.g., FH^+^  > FH^−^  > nFH) may indicate key familial traits that confer susceptibility but only lead to ASD when sufficiently severe, through interaction with separate liabilities, or when exacerbated in the context of developmental or stochastic effects. Familial traits detectable in infancy also may be particularly important for defining endophenotypes because they are less likely to be impacted by compensatory behavioral or brain processes in unaffected relatives. For example, behavioral differences in unaffected first-degree relatives of autistic individuals may not be as severe or detectable in later childhood or adulthood due to compensatory processes used to mask perceived challenges, or atypical maturational trajectories that converge with neurotypical patterns later in development [75, 76].

Additional examples of the power of family study designs for advancing gene discovery related to neurodevelopmental disorders can be found in adult psychiatry. For example, as described above, BSNIP takes a unique approach to family genetic research by developing hypothesis-driven and data-driven endophenotypes associated with psychosis that cut across diagnostic boundaries, deriving multi-level endophenotype factors that separate biologically separable clusters of patients, or “biotypes” [77, 78]. These biotypes do not align with diagnostic categories of DSM but are characterized by greater homogeneity of neural features and higher levels of intra-familial trait aggregation than DSM diagnostic categories [78]. Analyses of these biotypes in family genetic studies offer significant promise for gene discovery, and greater power than case-control studies using classification strategies that have less grounding in biology. Analogous approaches have yet to be leveraged in ASD, but such strategies could greatly increase power for detecting the additive, complex genetic substructures that contribute to autistic traits for the majority of affected individuals.

While family studies are central in the search for endophenotypic traits useful for defining etiological mechanisms associated with familial ASD (as opposed to ASD caused by de novo variants or environmental factors), their limitations also should be considered in the context of research aimed at understanding heritable influences. Family study methods provide only necessary, but not sufficient evidence that select traits are inherited. More specifically, family study designs are not capable of quantifying the impacts of shared environments and are thus not able to directly inform estimates of heritability. Family designs that focus on traits already shown to be highly heritable or less likely to be strongly influenced by social modeling or other environmental influences (e.g., social determinants of health such as socio-economic status or educational quality) may provide greater leverage in advancing endophenotype discovery.

Trait heritability is best demonstrated through twin studies examining concordance in monozygotic (MZ) and dizygotic (DZ) twins raised in the same family environments. Based on the assumption that MZ twins share approximately double the proportion of genetic material as DZ twins, greater similarity (i.e., concordance) between MZ relative to DZ pairs on select traits serves as an index of variation attributable to genetic inheritance rather than shared environment. Large-scale twin studies offer rich information on the heritability of individual traits that may directly guide analyses of candidate endophenotypes for clinical conditions or dimensions and guide interpretation of studies examining autistic individuals and their unaffected family members. For example, basic attentional and sensorimotor traits implicated in ASD each show high levels of heritability based on population twin studies and thus offer strong candidates for endophenotype discovery (e.g., [79, 80]). Importantly though, a series of twin studies examining eye-tracking data also has documented high levels of heritability in more complex processes including visual exploration during processing of social [80] and non-social scenes, as well as executive functions such as visual disengagement [81] and behavioral response inhibition [82]. Each of these behaviors has been implicated in ASD through either case-control or family study designs, suggesting that they may represent promising endophenotypes sensitive to inherited variation. Studies focused on the familial co-segregation of strongly heritable traits in ASD optimize the strengths of both family and twin designs for endophenotype discovery [79–82].

Twin studies have proven invaluable for developing new knowledge on the heritability of ASD and autistic traits. For example, twin studies consistently have indicated that ASD represents perhaps the most heritable behaviorally defined condition identified in the DSM [83–85]. They also have highlighted several key considerations in the analysis of trait inheritance patterns associated with ASD. First, heritability estimates show significant variation across studies (37–90%; [86–89]) due to multiple features, including the complexity of genotype-environment interactions, and the strong influence of measurement differences on the classification of individuals. For example, Colvert et al. [88] found that additive genetic effects explained 76–95% of diagnostic covariance when classifications were based on the Developmental and Well-Being Assessment and Autism Diagnostic Observation Schedule, but only 56% of covariance when classification was based on the Autism Diagnostic Interview–Revised (ADI-R). Second, evidence that trait concordance may be substantially lower in autistic relative to non-autistic twins implicates greater sensitivity to stochastic influences during development. Conducting the first known quantitative analysis of twin-twin severity of autistic traits, Castelbaum and Constantino [90] documented high levels of diagnostic concordance (96%) but low levels of autistic trait concordance across three different samples of autistic MZ twins (*R*^2^ < 0.1). Trait concordance was high in non-autistic population control MZ twins (*R*^2^ = 0.6) indicating that trait heritability estimates from non-autistic twins may not be directly translatable to ASD populations, and that environmental influences on trait outcomes and development may be profound.

In summary, family and twin studies each provide critical, complementary information for establishing endophenotypic traits associated with ASD. While family designs, including trio, quad, and infant sibling methods, each can yield important new insights into traits that “run in families”, they are not able to directly index heritability of candidate traits. Instead, heritability estimates from twin studies are important for understanding inheritance patterns and parsing environmental and genetic contributions to trait outcomes. Twin studies also are limited by difficulties ascertaining sufficient samples of autistic twins and evidence that heritability estimates from non-autistic twins may not be directly applicable to autistic twins. Large-scale twin studies that characterize carefully selected continuously distributed traits in the general population represent a promising future direction. More specifically, focus on analyses of early emerging heritable traits less influenced by stochastic or environmental processes will be important for identifying endophenotypic traits sensitive to inherited genetic processes.

## Candidate endophenotypes and trait domains associated with ASD

The majority of traits examined in family studies of ASD have been measured at the behavioral level. It is important to note that endophenotypes originally were differentiated from behavioral traits based on their “internal” and “unobservable” qualities allowing them to be objectively measured and suggesting they lie more proximal to gene action [26, 91, 92]. Based on this premise, it has been argued that familial behavioral traits should be considered separately as intermediate phenotypes [92]. This distinction is important in the context of findings that behavioral and cognitive dimensions studied as familial traits in ASD also have highly complex genetic architectures, suggesting their added value in advancing knowledge of pathogenic processes relative to clinical symptoms may be limited [27, 92, 93].

Despite these caveats, we propose that familial behavioral traits are important to integrate in family genetic studies because they can be leveraged to focus the search for associated “internal” traits closer to genetic substructures. Studies of core autistic traits represent important examples of how understanding familial behavioral phenotypes may guide the search for endophenotypic traits. For example, considerable evidence shows that first-degree relatives of autistic individuals present mild traits qualitatively similar to those found in their autistic family members, justifying the definition of a “broader autism phenotype” (BAP) characterized by social aloofness, pragmatic communication difficulties, and a rigid personality style [94, 95]). Notably, a stepwise pattern of BAP trait loading has emerged for multiplex, simplex, and adoptive parents of autistic children [96, 97] indicating that trait burden reflects an aggregation of ASD-related genetic liability, and that dosage of genetic influences is greater in families with recurring ASD [98, 99]. Consistent with this hypothesis, Lyall et al. [100] documented that child ASD risk is increased by 85% when both parents show elevated autistic traits. Collectively, these findings provide strong support that analysis of BAP traits across family members holds significant power for determining familial ASD processes and informing genetic models [94, 95, 98–100].

In principle, identifying endophenotypic traits associated with BAP behaviors and those features closer to molecular and neurodevelopmental mechanisms contributing to ASD will increase resolution for detecting etiological pathways. This process of mapping ETDs by linking behavioral traits back to their genetic origins aims to define the full chains of causality contributing to traits that may collectively constitute ASD. To illustrate how this approach may enhance resolution for detecting genetic substructures, we integrate findings from case-control and family designs assessing the neurodevelopmental substrates of multiple behavioral traits associated with ASD. Our list of candidate ETDs is not intended to be exhaustive. Instead, we focus on discrete behaviors that are highly quantitative, have been studied across multiple levels, and also have shown promise for clarifying etiological processes based on studies of their heritability, familiality in ASD, underlying biology, and potential for back-translation to model systems (see Table 1).

**Table 1:** Summary of evidence for candidate ASD-related endophenotypic trait domains (ETDs) across multiple levels of analysis. ETDs described here represent some of the most promising targets for ASD research on endophenotypes based on accumulating evidence showing targeted phenotypes are quantitative, translational, present in unaffected relatives of autistic individuals, and measurable across multiple units of analysis. This table is not intended to be a comprehensive list of candidate ETDs associated with ASD but represents perhaps the most promising ETDs based on existing data

**Level of analysis**	**Endophenotypic trait domain (ETD)**			
	**Social gaze**	**Language/Communication**	**Executive function**	**Sensorimotor behavior**
Neuropsychological and Behavioral	Relative to age-matched neurotypical controls, autistic children show reduced attention to eye regions of faces [101, 102]; Autistic adolescents and adults show reduced attention to faces [103, 104] Infants later diagnosed with ASD show declining rates of gaze shifts towards the eye regions of others between 2–6 months resulting in reduced overall attention to faces relative to neurotypical peers [102] Parents and siblings of autistic individuals show atypical social gaze [51, 105], and parents with broader autism phenotypic (BAP^+^) characteristics show decreased gaze towards social stimuli in complex scenes relative to population controls and parents of autistic individuals who do not show BAP features (BAP-; [105, 106])	Autistic individuals show higher mean pitch, greater pitch variability, and longer voice duration in autistic individuals relative to controls [107, 108] Pain cries from 6-month-olds with a family history (FH) of autism are higher and more variable in pitch than infants with no FH (nFH) [46] Cries of 15-month-old FH toddlers show higher frequencies [42] and are shorter in duration than nFH toddlers [42, 109] FH infants later diagnosed with autism (FH^+^) have higher fundamental frequencies than FH infants who do not develop autism (FH^−^); FH- infants have higher fundamental frequencies than infants without FH of autism, (FH + > FH- > nFH) [42] Parents of autistic children show some overlap with their offspring in suprasegmental speech, including greater variability in frequency, especially for mothers showing high levels of BAP features [108]	Across large age ranges, autistic individuals show increased rates of errors during tests of behavioral response inhibition (e.g., stop signal task, antisaccades) associated with more severe clinically rated RRBs [67, 110–112] Compared to respective controls, autistic individuals and their first-degree relatives show a reduced ability to shift behavioral responses during neuropsychological tests of cognitive flexibility (e.g., probabilistic reversal learning, set shifting) [113–115] Autistic individuals and their parents show increased rates of errors on probabilistic learning and stop signal tasks; reversal learning errors were more severe in BAP + parents and their children relative to BAP- families; cognitive flexibility and inhibitory control error rates were increased in autistic children of BAP+ parents and associated with more severe ASD symptomatology [67]	Autistic individuals show increased force variability in precision grip manual motor output relative to neurotypical controls during a steady state force maintenance task [116] Infants with different ASD-risk status show stepwise patterns in quantitative measures of reach-to-grasp movements such that FH + infant siblings had worse scores on the Skilled Reaching Rating Scale than FH- and nFH infants [117] Infants with different ASD-risk status show stepwise patterns in behaviorally coded postural control behaviors (e.g., lying, supported or unsupported sitting, resting on all four limbs, supported and unsupported standing) indicating delayed trajectories of primary postural positions in FH + infants, relative to FH- with language delay, FH- without language delay and nFH [FH + < FH- with language delay < FH- without language delay = controls; [118] Parents and siblings of autistic individuals show reduced accuracies of saccadic eye movements, reduced smooth pursuit eye movement velocity during closed-loop phases and lateralized reductions of smooth pursuit eye movements during the open-loop phase implicating reduced lateralization of sensorimotor behavior [119] First-degree relatives of autistic individuals showed similar open-loop pursuit gain for rightward and leftward movements, whereas same-age controls showed greater gain for rightward relative to leftward movements implicating left hemispheric dominance [119]
Brain (cellular, circuit and network levels)	Autistic individuals consistently show prolonged N170 latencies to images of faces suggesting delayed or less automated processing of facial information [120, 121] Autistic individuals and their unaffected siblings also demonstrate a reduced difference in N170 amplitudes between inverted and upright faces compared to controls suggesting reduced specialization of neural processing of facial information that is familial [122] First-degree relatives of autistic individuals show diminished right lateralization of N170 while viewing static images of faces relative to respective neurotypical control groups suggesting familial patterns of altered developmental specialization of select social brain networks [123, 124] Autistic individuals and their siblings show reduced functional activation to faces in amygdala, fusiform face area, left frontal gyrus, right middle prefrontal cortex (PFC), left posterior PFC, left dorsomedial PFC, and temporal poles relative to controls [125] Parents of autistic individuals show increased functional activation in fusiform face area and amygdala compared to control parents; BAP+ parents have greater right fusiform gyrus activation than BAP- and control parents [126]	Autistic individuals show less stable frequency following responses (FFRs) to speech sounds relative to non-autistic peers suggesting greater levels of variability in processing auditory information [127] Autistic individuals and their parents show reduced auditory P1 amplitudes relative to age-matched controls, reflecting less robust detection of changes in pitch during auditory feedback [128]	Autistic individuals show reduced activation in both prefrontal cortex and ventral striatum when attempting to shift to a new behavioral response after removal of reinforcement for a previously correct response [129] Autistic individuals show atypical activation of anterior cingulate during an antisaccade task requiring participants to look away from suddenly appearing targets [130, 131] Autistic individuals show reduced activations across frontal and parietal eye fields during antisaccades relative to non-autistic controls [132]	Autistic individuals show atypical activation of premotor and parietal cortex during manual motor behaviors relative to controls [133, 134] Autistic individuals show reduced functional connectivity of inferior parietal lobule and cerebellum and atypical age-related differences in cerebellar-cortical functional connectivity during manual motor behavior [133] Autistic individuals show right lateralization motor circuit connectivity during task-free functional MRI compared to controls associated with more severe clinically rated motor impairments [135] Lesions of posterior cerebellar vermis in non-human primates impair feedback mechanisms supporting saccade accuracy [136]; persistent deficits in modulating accuracy across repeated events/trials highlight a critical role in error feedback correction that also is seen in autistic individuals implicating cerebellar modulation of brainstem circuits [137]
Molecular and genetic	Non-human primates with TALEN-edited MECP2 mutations show a preference for social over nonsocial stimuli and look to conspecific faces exhibiting aggressive and submissive expressions for shorter durations than neutral expressions [138] Primate offspring of maternal immune activation ASD models show multiple gaze differences relative to control animals, including longer latencies to fixate on the eye region of conspecific faces, fewer fixations directed at the eyes, and less fixation time on the eyes [139] Multiple mouse models of ASD show reduced social approach relative to wild-type (WT) mice [140, 141]; Rescue of social approach deficits with R-Baclofen in BTBR mice [142] and mice with 16p11.2 microdeletion [143] suggest reduced GABA_B_ function associated with reduced social attention in preclinical models of ASD	SHANK3 KO mice and other preclinical models of ASD show higher peak frequencies and reduced modulatory abilities in vocalizing relative to wild type mice [144, 145]	BTBR mice exhibit elevated rates of errors during reversal learning [146, 147] Reversal learning deficits in BTBR mice were rescued with administration of an adenosine A_2a_ receptor agonist and 5HT_2a_ receptor antagonist in dorsomedial striatum [148, 149]	DVL1-modified and FMR1 KO mice show atypical sensorimotor gating [150, 151] Rescue of cerebellar Purkinje cell function and atypical gait and balance phenotypes was achieved through the mTOR pathway [152, 153] In a valproic acid exposure mouse model of ASD, mice demonstrated specific cell loss in motor cortex and cerebellum and motor impairments that were associated with Purkinje cell loss in Crus I [151]

*FH* Family history of ASD (FH^+^: family history of ASD with later ASD diagnosis, FH^−^: family history of ASD without later ASD diagnosis), *nFH* No family history of ASD, *KO* Genetic knock-out, *BAP *Broad autism phenotype (BAP+, BAP−)

### Social gaze

Social impairments have served as the pathognomic features of ASD since Kanner’s original case studies [154]. They include a broad class of behavioral issues ranging from differences in basic attentional allocation to social information to more complex cognitive difficulties in the processing of social cues and development of social relationships (e.g., [101, 155]). The complex nature of many social behaviors that are impacted in ASD highlights challenges in establishing their underlying biology and therefore identifying endophenotypic traits. Quantitative studies of social attention have served to deconstruct complex behaviors into simpler cognitive processes that offer more potential for back-translation to preclinical models and may represent simpler genetic substructures than complex social phenotypes. For example, social gaze toward faces represents a highly quantitative biomarker that appears to show familial patterns. Importantly, eye tracking of social gaze can be done in studies of wide ranges of ages as demonstrated by multiple studies documenting non-linear changes in social gaze as early as the first weeks of life through adulthood (e.g., [101]). Leveraging similar measurement approaches, foundational work from Jones and Klin indicated that infants later diagnosed with ASD show declining rates of gaze shifts toward the eye regions of others between 2 and 6 months resulting in reduced overall attention to faces relative to neurotypical peers [102]. These findings are consistent with separate studies documenting reduced attention to faces in autistic adolescents and adults [103, 104]. Reduced eye gaze is strongly associated with the severity of autistic traits suggesting that this highly quantitative trait may represent a key endophenotype related to liability for autistic traits or ASD affectation.

Family studies provide support for social gaze as a useful endophenotypic trait associated with ASD. Studying non-autistic twins, Constantino et al. [79] demonstrated high levels of heritability for infant gaze to eye and mouth regions. MZ twins also showed high concordance of the duration and direction of saccadic eye movements when viewing social scenes, consistent with separate studies documenting high levels of heritability for social gaze in more complex social environments [80]. Social gaze differences also appear to be strongly familial in ASD [80]. Atypical social gaze is seen in parents [105] and siblings of autistic individuals [51], and parents with BAP characteristics show decreased gaze toward social stimuli in complex scenes relative to population controls and parents of autistic individuals who do not show BAP features [105, 106]. These findings suggest that atypical social gaze is associated with core autism behavioral traits in unaffected family members and may serve as a quantitative link between social traits of ASD and their inherited biological underpinnings.

Quantitative EEG/ERP studies of social gaze in ASD offer insights into the neural substrates of social gaze phenotypes. The N170 peaks 150–200 ms after stimulus onset and is sensitive to the visual presentation of social stimuli including faces [156]. During viewing of faces, the N170 is thought to index early structural encoding of facial features and face categorization [157, 158]. In non-autistic individuals, the latency of the N170 ERP is shorter to faces than to houses or other objects [120] suggesting increased automaticity of percept processing. The N170 to faces also typically is right lateralized implicating hemispheric specialization for processing facial information [159]. Delayed N170 latencies have been consistently documented in autistic individuals compared to non-autistic controls (e.g., [120, 121]), and they appear to be associated with reduced memory for faces [160]. Both autistic individuals and their unaffected siblings also demonstrate a reduced difference in amplitudes between inverted and upright faces compared to controls suggesting reduced specialization of neural processing of facial information that is familial [122]. Parents [123] and infant siblings of autistic individuals show diminished right lateralization of N170 amplitudes associated with more severe social-communication impairments and sensory symptoms further indicating that developmental specialization of select social brain networks represents a familial neural endophenotype associated with core autistic characteristics [124].

While a myriad of MRI studies have identified structural and functional brain alterations [104, 161, 162] associated with atypical social gaze in ASD implicating prefrontal and temporo-parietal circuits as well as amygdala nuclei (for reviews, see [163, 164]), only a small number of MRI studies have examined neural correlates of social gaze in unaffected family members. During face viewing, parents of autistic individuals irrespective of BAP status demonstrated increased functional activation of the fusiform face area and amygdala [126] suggesting that increased activation in these regions associated with social gaze processing reflects ASD-familial status; however, parents with the BAP showed more severe hyper-activation of right fusiform gyrus than parents without the BAP and controls. Consistent with this findings, both autistic individuals and their unaffected siblings show reduced frontal (i.e., left superior frontal gyrus; right middle, left posterior, left dorsomedial PFC) and temporal (i.e., temporal pole, fusiform face area) activation during face viewing relative to controls [125] indicating alterations of neurodevelopmental processes supporting the functional specialization of brain networks involved in facial processing represent strong candidate endophenotypes for elucidating familial and perhaps inherited trait processes.

Multiple separate preclinical studies also have been conducted to understand cellular, physiological, and molecular genetic mechanisms of different social traits associated with ASD. Current challenges in back-translation of ASD-related social differences include limits to mimicking complex social behaviors in model systems such as the rodent models that often are used in genetic knock-out (KO) studies. Despite these challenges, studies of non-human primates have provided some insight into cellular and molecular processes associated with social gaze behavior. Chen and colleagues found that non-human primates with TALEN-edited *MECP2* mutations exhibited a preference for social over nonsocial stimuli and looked to conspecific faces exhibiting aggressive and submissive expressions for shorter durations than neutral expressions [138]. Based on findings implicating maternal immune system activation in autistic liability, non-human primate maternal immune activation models have also been studied to understand basic physiology of social gaze and relationships with autistic traits. During viewing of facial images of conspecifics, offspring of maternal immune activation models showed multiple gaze differences relative to control animals, including longer latencies to fixate on the eyes, fewer fixations directed at the eyes, and less fixation time on the eyes [139]. Studies of non-human primates defining cellular and synaptic processes supporting social gaze also may be integrated to clarify cellular and molecular mechanisms of social gaze differences in ASD and their genetic underpinnings. Non-human primate studies have documented selective firing of lateral interparietal neurons during social gaze and gaze shift behavior [165] and increased firing of amygdalar cells during viewing of eyes and direct eye contact [166].

In contrast to non-human primate studies, mouse genetic studies allow for interrogation of gene-specific effects and downstream molecular and electrophysiological traits. Yet, they are limited in the extent to which behavioral phenotypes can be translated to inform understanding of complex social traits in humans given limited homology of brain and behavior between humans and rodents. For example, common behavioral assays for rodent models of social traits in ASD include nose-to-nose sniffing, pushing or crawling, time spent in a chamber with (or at a partition next to) another mouse versus alone, partner preference, and social transmission of food preference [167]. Most of these behaviors do not closely mirror naturalistic social behavior in humans; however, social approach, withdrawal, and recognition are cross-species social behaviors that may be more easily translatable from rodent models to humans. Multiple mouse models of autism have documented reduced social approach relative to wild-type (WT) mice, suggesting multiple gene variants associated with ASD may contribute directly to diminished interest in or increased resistance to social interaction [140, 141]. Findings that the selective GABA_B_ enantiomer R-Baclofen reverses deficits in social approach in BTBR mice [142] and mice with 16p11.2 microdeletions [143] suggest that atypical GABA signaling may underpin social approach differences in some autistic individuals, though R-Baclofen has shown variable effects on social difficulties in autistic individuals. Integration of these preclinical studies therefore offers important traction for understanding molecular and cellular processes that may contribute to social impairment in autistic individuals, though these studies also highlight the significant challenges in translating model system findings regarding the pathophysiology of social difficulties to humans.

Together, data across behavioral, cognitive, and brain system levels suggest that social gaze traits and their neural substrates comprise a promising ETD linked to the pathognomonic features of ASD. Separate social behavioral traits associated with ASD also warrant further study. For example, atypical functional activations of fusiform gyrus, superior temporal sulcus, middle frontal gyrus, and amygdala during biological motion processing have been identified in both autistic individuals and their unaffected siblings, including compensatory activations seen specifically in unaffected siblings [49]. The extent to which biological motion processing differences and associated alterations in functional brain development relate to differences in social gaze or represent a unique ETD in ASD remains unclear. Histological analyses of cellular features and gene expression in targeted brain regions of autistic individuals and their family members also will be important for determining the molecular substrates of familial social gaze differences.

### Language and communication

Autistic individuals show a range of language and communication challenges including reduced receptive and expressive language abilities as well as difficulties in pragmatic forms of communication [168]. Differences in the suprasegmental aspects of speech, including prosody (e.g., intonation, rate, and rhythm of speech) also are common. Atypical intonation, rate, and rhythm of speech appear to be familial, suggesting they represent promising targets for establishing endophenotypes useful for understanding genetic pathways associated with autistic traits and liability [20, 168, 169].

Meta-analytic data indicate that autistic individuals exhibit higher mean pitch, greater pitch variability, and longer voice duration relative to controls [107]. These features have been highly reliable for distinguishing autistic from non-autistic individuals [107, 108]. Differences in suprasegmental components of speech among autistic individuals are also evident in tonal languages which use pitch to convey not only pragmatic information, but also word meaning [170]. For example, Cantonese speaking autistic children and adults show less robust prosodic encoding compared to controls, suggesting that difficulties processing suprasegmental features of speech in ASD persist into adulthood despite years of experience with a tonal language [170].

Measuring non-speech vocalizations also is important for understanding language and communication development across the lifespan in autism. For example, infant siblings of autistic children show differences in vocal properties that emerge early in ontogeny, including atypical prosody during crying in infancy [42, 46]. At 6 months, pain cries from FH infants are higher and more variable in pitch than those of nFH peers [46]. Similarly, Esposito and colleagues found that cries of 15-month-old FH toddlers showed higher frequencies [42] and were shorter in duration than nFH toddlers [42, 109]. Importantly, FH^+^ infants had higher fundamental frequencies than FH^−^ infants, who had higher fundamental frequencies than nFH infants (FH^+^ > FH^−^ > nFH) [42]. Parents of autistic children show some overlap with their offspring in suprasegmental speech, including greater variability in frequency driven specifically by mothers showing high levels of BAP features [108]. These findings suggest differences in suprasegmental aspects of speech that may represent endophenotypic traits in a select subset of families and therefore may serve as important targets for parsing etiological heterogeneity [171, 172]. Large-scale quantitative family studies assessing interactions of maternal autistic traits and suprasegmental aspects of speech hold promise for understanding inherited genetic processes contributing to ASD in select clusters of families.

Suprasegmental features of speech represent important targets for studies of endophenotypes because relevant measures also can be translated to neuroimaging environments and across species. Studies of neurophysiological processes associated with speech differences in ASD have provided important information on candidate endophenotypic traits. The frequency following response (FFR) is an early auditory-evoked potential which marks how sounds at the frequency of natural speech and their harmonics are encoded in relevant cortical pathways [173–175]. The degree to which frequency of the stimulus is reflected in the FFR indexes the integrity of the auditory pathway for encoding early features of acoustic stimuli [175]. Importantly, FFR responses can be leveraged across the lifespan to understand neural processing of sound, especially since this neurophysiological measurement is a customary practice in newborn hearing screening. Autistic individuals show less stable FFR responses to speech sounds relative to non-autistic peers suggesting greater levels of variability in processing auditory speech information [127]. Differences in the neural processing of sounds may underlie difficulties monitoring, adjusting, or matching aspects of speech like tone, rate, and rhythm, which may in turn affect higher-order features of language like prosody and pragmatics. Additionally, during a task of vocal production with dynamic auditory feedback, autistic individuals and their parents showed reduced auditory P1 amplitudes relative to age-matched controls, reflecting less robust detection of changes in pitch during auditory feedback [128]. Diminished ability for feedforward vocal control in contexts requiring audio-vocal integration may be a mechanism of prosodic differences in ASD and may also be reflective of disrupted neuromotor control of speech.

Back-translation of language and communication traits into preclinical models represents a major challenge given the unique language and communication abilities of humans and non-human primates. Still, analysis of suprasegmental speech offers some opportunity for translational research that may be informative for understanding genetic and molecular substrates of impairments in ASD. For example, differences in the acoustic properties of pup vocalizations can be measured in rodent species and appear to be selectively disrupted by KO of multiple ASD-related genes. More specifically, higher peak frequencies and reduced modulatory abilities of vocalizations have been demonstrated in mouse models of ASD [144], including higher peak frequencies and reduced modulatory abilities in SHANK3 KO, mirroring results found in autistic individuals [145]. Separate non-human primate models of ASD may be leveraged to characterize qualities of vocalizations associated with ASD to help bridge pre-clinical rodent findings to brain system, neuropsychological, and behavioral level communication traits associated with ASD.

Language-related endophenotypes separate from suprasegmental speech characteristics also exist (e.g., pragmatic communication features, expressive vocabulary). Fewer studies have examined the familiality and neural substrates of these distinct language and communication traits owing to multiple considerations, including their relative complexity (e.g., pragmatic communication) and limited suitability for imaging environments sensitive to oromotor movements (e.g., functional MRI). Studies of language-related endophenotypes also are hindered by difficulties assessing the full range of language impairments in ASD, including both non-speaking and fluent individuals. Assessment of neurophysiology may be particularly challenging with autistic individuals who show severe impairments in processing or expressing language, as these individuals may not process instructions or task demands. Similarly, individuals with severe expressive language disability may show limited ability to respond to task demands, or rates of echolalia that may make it difficult to obtain adequate naturalistic language samples [176]. The growing use of augmentative and alternative communication (AAC) strategies may also introduce questions of comparability between expressive language in written and spoken modalities. These challenges have contributed to a dearth of studies including the full range of language traits in studies of ASD, confounding progress in determining etiological processes. There is a critical need to develop strategies to integrate individuals who are minimally- or non-speaking and, where possible and appropriate, utilize compound tasks that involve both direct measurement of language and simultaneous tracking of biobehavioral indices (e.g., imaging, neurophysiology, eye tracking, oromotor movements).

### Cognitive control

Executive functions represent a diverse range of cognitive abilities supporting the regulation of thought and action, including the abilities to inhibit dominant responses, update working memory representations, and flexibly shift behavior or cognitive strategies in response to changes in environmental demands. Consistent with their diversity, the genetic substrates of executive abilities are complex. Executive functions show high levels of heritability involving a common genetic “factor” as evidenced in the analysis of twin concordance (e.g., [177]). While these data implicate common, likely polygenic processes contributing to high levels of heritability for executive functions, different executive abilities also appear to be modified by separable genetic influences that contribute to diverse estimates of heritability for individual functions [177, 178]. These results are consistent with studies documenting modest covariation of different executive abilities and the overarching conceptualization that executive processing is a multi-dimensional set of cognitive operations [179, 180]. The genetic structures of executive abilities also appear to show complex interactions with general cognitive abilities, showing both covariation and high levels of heritability that are distinct from genetic influences on IQ, processing speed, and visuospatial abilities [177, 178]. These findings collectively indicate that while executive abilities involve multiple diverse functions, heritability is high and trait inheritance likely reflects common pathways that also can be modified by separable genetic influences that shape individual executive outcomes.

Despite findings that executive functions are among the most heritable psychological traits [177], and that they are consistently disrupted in autistic individuals [181], their familiality in ASD seldom has been examined. Behavioral rigidity, perhaps the most common trait characteristic of family members of autistic individuals [97], appears to involve alterations of executive processing, including reduced abilities to flexibly shift cognitive strategies and to inhibit prepotent behavioral responses [67]. For example, prior work by our group and others has demonstrated that reduced cognitive flexibility and behavioral response inhibition in autistic individuals each are associated with more severe clinically rated RRBs, including a strong need for sameness in the environment and in routines (i.e., insistence on sameness) and compulsions [110–112]. These findings together implicate cognitive inflexibility and reduced behavioral response inhibition as important cognitive trait markers associated with behavioral rigidity.

Similar cognitive traits have been implicated in unaffected family members. Reduced behavioral flexibility, characterized by a strong insistence on sameness and intense preoccupations, appears to co-segregate in families of autistic probands [182–184]. Studying a probabilistic reversal learning test in which individuals must shift a response selection away from a previously reinforced item after reinforcement contingencies have changed, Schmitt et al. [67] documented that unaffected parents of autistic individuals show an increased rate of regressing back to previously reinforced stimuli, or “regressive errors”, similar to findings from a previous study of autistic individuals performing the same reversal learning task [110]. These findings were consistent with prior studies documenting reduced cognitive flexibility on tests of set shifting in autistic individuals and unaffected first-degree relatives [113–115] but also extended this work to show that increased rates of regressive errors were specific to parents showing high levels of BAP features and their autistic children [67]. These results suggest that cognitive control traits are familial and that they may contribute to or interact with core autistic traits to increase ASD likelihood of affectation. These findings are particularly promising for defining multi-factorial endophenotypes (BAP features and cognitive control impairments) that may co-segregate and be useful for establishing more homogeneous subgroups, or biotypes, for family genetic research. Results also may help explain prior findings that some unaffected family members show relatively intact cognitive flexibility as subsets of families with an autistic family member but without parental BAP traits may have relatively preserved cognitive flexibility [185, 186]. Analyses of inhibitory control abilities among this same sample of autistic individuals and their parents showed increased error rates in parents and autistic individuals relative to age-matched controls, though deficits appeared to be independent of parental BAP status [67]. Together, these findings suggest that cognitive flexibility and inhibitory control impairments each may represent important familial endophenotypes associated with RRBs in probands and behavioral rigidity in parents, and that their analysis could help parse etiologic heterogeneity by identifying more cognitively homogeneous subsets of families.

Tests of cognitive flexibility (probabilistic reversal learning) and inhibitory control (stop signal and antisaccade) constitute important measures for ETD studies because each can be adapted to neuroimaging environments to examine underlying brain functions (e.g., [129, 187, 188]). Reversal learning is supported by neural systems including middle frontal gyrus, posterior parietal cortex, striatum, and midbrain nuclei [189]. Using fMRI, D’Cruz et al. [129] documented reduced activation in both prefrontal cortex and ventral striatum in autistic individuals relative to controls during trials in which participants needed to shift their response set. Reduced activation in frontal cortex implicates problems in decision-making and response planning, while atypical activation of ventral striatum suggests limited processing of reinforcement cues, as has been demonstrated more broadly in ASD [190, 191]. These processes, and their integration, are essential for flexible behavior. Alterations in these systems may therefore contribute to a rigid adherence to preferred behavioral patterns in autistic individuals.

Cognitive/behavioral flexibility also is a promising target for studies of ETDs in ASD because of the potential for back-translation to model systems. Probabilistic reversal learning tests in particular have proven to be highly translational strategies for studying neurophysiologic, cellular, and molecular mechanisms of cognitive/behavioral rigidity across species. For example, using a probabilistic reversal learning paradigm similar to that described above in clinical studies of autistic individuals, Ragozzino and colleagues have conducted a series of studies documenting elevated rates of errors in multiple mouse models of ASD [e.g., BTBR mice, mice reared in conditions of high maternal stress or maternal exposure to a selective serotonin reuptake inhibitor (SSRI)] [146, 147]. They also have demonstrated rescue of reversal learning deficits in mouse models with administration of an adenosine A_2a_ receptor agonist and a 5HT_2a_ receptor antagonist in dorsomedial striatum implicating selective receptor targets for drug development as well as gene expression studies focused on cognitive/behavioral rigidity in ASD [146–149].

Studies of inhibitory control show similar potential for translation across behavioral and imaging environments in ASD studies and for back-translation in lower-order species. Using tests of antisaccades, or the ability to consistently suppress prepotent oculomotor responses, multiple fMRI studies have detailed neural networks supporting top-down control of reactive behaviors, including frontal and parietal eye fields, anterior cingulate cortex, middle frontal gyrus, and dorsal striatum [187]. Increased rates of antisaccade errors have been repeatedly documented in autistic individuals [130, 131, 187, 192, 193]. and their unaffected first-degree relatives [111], and shown to be associated with reduced activations across frontal and parietal eye fields [132] that are important for generating motor plans [194]. Atypical activation of anterior cingulate also has been observed during antisaccades in ASD, suggesting deficits in response monitoring processes central to modifying behavior in response to external contingencies [130, 131]. Antisaccade tests frequently have been examined in non-human primates to determine the cellular processes that support inhibitory control (e.g., [195, 196]), and separate tests of behavioral response inhibition have been developed to examine these processes in rodent models (e.g., [197]). Parallel human and model system studies similar to those examining cognitive flexibility described above may help elucidate distinct genetic and molecular processes contributing to familial deficits of inhibitory control in ASD.

### Sensorimotor control

Sensorimotor functions frequently are impaired in ASD affecting a range of behaviors and effector systems including oculomotor, vestibulo-motor, and skeletomotor processes [116, 198, 199]. Sensorimotor impairments in ASD also emerge early in development, perhaps earlier than primary social-communication and cognitive/behavioral traits [200–207]. Sensorimotor behaviors represent highly promising targets for endophenotype research because they can be decomposed into quantitative motor control processes subserved by discrete neural systems. For example, while a broad range of sensorimotor behaviors have been shown to be affected in ASD (e.g., [198, 199, 208]), converging evidence indicates a reduced ability to integrate multi-sensory feedback to guide ongoing behaviors resulting in increased variability and regularity of motor output [116, 209–211]. Brain systems supporting sensory feedback control during motor behavior have been well-delineated via non-human primate, human lesion, and basic neuroimaging studies and include temporo-parietal and occipital circuits involved in sensory processing and integration, premotor and primary motor cortices involved in planning and executing motor commands sent to the periphery, and cerebellar circuits involved in modulating motor commands to motor cortex based on sensory feedback error information relayed via cortical-pontine circuits (e.g., [212–214]). Cortical and cerebellar circuits involved in sensory feedback control of motor behavior each have been implicated in ASD via anatomical MRI studies [215] and post-mortem brain studies [216–219]. These results highlight the strong promise of investigating sensorimotor behavioral dysfunctions across motor control, brain system, and cellular levels for mapping ETD pathways associated with ASD.

Multiple sensorimotor behaviors, including sensory feedback guided precision manual motor behaviors and sensorimotor sequence learning, each show high levels of heritability in twin studies [220]. Analyses of the neural substrates of these sensorimotor behaviors also show strong inherited genetic contributions. More specifically, structural features of unimodal (sensory/motor) cortical regions show reduced variability and increased heritability relative to structural characteristics of heteromodal association networks involved in more complex cognitive operations [221]. These findings together provide evidence that the study of sensorimotor traits and their neurodevelopmental substrates may offer significant leverage for identifying ETDs associated with inherited autistic traits [220–222].

The hypothesis that sensorimotor traits represent promising candidate endophenotypes is supported by studies showing that sensorimotor impairments in ASD also may manifest in first-degree family members. Examining multiple oculomotor behaviors, we previously documented that impairments seen in autistic individuals also were present in their unaffected biological parents and siblings, including reduced accuracies of saccadic eye movements, reduced smooth pursuit eye movement velocity during closed-loop phases, lateralized reductions of smooth pursuit eye movement velocity during the open-loop phase (i.e., the initial period of smooth pursuit that precedes the availability of sensory feedback input due to afferent delays), and atypical lateralization of procedural learning of saccadic eye movements [119]. Each of these oculomotor differences parallels findings from prior studies of autistic probands [223–226] suggesting oculomotor abnormalities may track in families of autistic individuals. Studies of infant siblings of autistic children also highlight strong familial patterns consistent with the hypothesis that different sensorimotor abilities may represent promising endophenotypes. Infant sibling studies using standardized tests of fine and gross motor developmental abilities have indicated that motor skills are predictive of language outcomes [62, 201, 227] and autistic traits in FH infants [205, 228–230]. A stepwise pattern has been documented in which sensorimotor impairments are more severe in FH^+^ siblings relative to FH^−^ siblings who show differences compared to controls [62, 205]. It should be noted that infant sibling studies of sensorimotor behaviors also have yielded inconsistent results owing to multiple factors, including variance related to the abilities tested, diversity of the measures used to test early sensorimotor development, and developmental heterogeneity across the samples studied. Studies using behavioral coding or wearable sensors suggest that more consistent patterns of familiality may be detectable with quantitative measures relative to standardized behavioral assessments that aggregate categorical items (e.g., Mullen Scales of Early Learning). For example, stepwise patterns were seen in infant siblings (FH^+^  > FH^−^  > nFH; [117]), and during postural control, familial patterns were evident that varied according to whether FH siblings were diagnosed with ASD or language delay (FH^+^  < FH^−^ with language delay < FH^−^ without language delay = controls; [118]). Together, these findings offer strong support that separate sensorimotor behaviors may serve as endophenotypic traits representing early emerging inherited factors associated with autistic traits and related developmental issues (e.g., language delay). Separate sensorimotor behaviors do not show familial patterns (e.g., quantity of movement [231]) indicating that only a circumscribed set of sensorimotor behaviors will offer power as endophenotypic traits indexing heritable pathways associated with ASD.

The multiple familial oculomotor differences identified in studies of autistic individuals and first-degree relatives each implicate separate neurophysiological processes. The accuracy of saccadic eye movements, or rapid, ballistic shifts in eye gaze, is guided by frontal and parietal eye fields and modulated on a trial-to-trial basis by cerebellar-brainstem circuits that act to reactively adjust output precision according to error feedback information [232]. Smooth pursuit eye movements include both closed- and open-loop phases separated by the extent to which sensory feedback is available to guide output precision. Reduced accuracy of closed-loop pursuit suggests atypical processing of error feedback information in cerebellum as well as prefrontal cortex and frontal eye field circuits involved in planning motor behavior. Consistent with these findings, multiple studies have documented atypical activation of premotor and motor cortex during motor behavior [133] and cerebellar-cortical functional connectivity during visuomotor behavior [133, 134] in autistic individuals. These sensorimotor data implicate familiality of atypical neural functioning within systems supporting motor planning, sensory feedback guided motor behavior. Findings of reduced lateralized dominance during open-loop smooth pursuit eye movements in autistic individuals and their first-degree relatives [119, 233] also are consistent with findings of atypical functional lateralization of motor circuits in autistic individuals during rest [135], reduced lateralized hand dominance [135], and results showing reduced lateralization of ERP N170 components during facial processing [234], to suggest reduced hemispheric specialization of functional brain networks may be a familial endophenotypic trait affecting multiple developmental abilities in autism. Analyses of how different sensorimotor endophenotypes co-vary across family members are needed to determine whether they represent separate ETDs associated with distinct genetic substructures [119, 133, 135, 232–234].

Sensorimotor behaviors also offer important advantages in the search for endophenotypes based on their translational nature. Motor control systems are largely preserved across different species allowing for direct back-translation. Oculomotor systems have been studied extensively in non-human primates advancing resolution of the circuits and cellular processes contributing to distinct behaviors. For example, Takagi et al. [136] documented that ablation of posterior cerebellar vermis impairs feedback mechanisms supporting saccade accuracy. Recovery of function is seen in primates, though persistent deficits modulating accuracy across repeated events/trials are evident, highlighting a critical role in error feedback correction that also is seen in autistic individuals implicating cerebellar modulation of brainstem circuits [137]. Subsequent analyses show that olivary climbing fibers synapsing with cerebellar Purkinje cells are necessary for feedback learning and may act as a crucial circuit for interrogation of ASD-related sensorimotor impairment [235].

Multiple mouse genetic models of ASD also show sensorimotor deficits, including deficits of coordination and increases in movement variability offering important leads for clarifying molecular mechanisms associated with sensorimotor and perhaps downstream behavioral differences in ASD [150, 236]. Another mouse model of ASD via valproic acid exposure produced selective cell loss in motor cortex and cerebellum, with a greater degree of cell loss in males than females. In addition, both male and female mice exhibited motor deficits that were associated with Purkinje cell loss in crus I of cerebellum [151]. Findings that rescue of cerebellar Purkinje cell function through the mTOR pathway also indicate that understanding of the molecular, cellular, and neurophysiological bases of sensorimotor impairments may guide new therapeutic strategies targeting familial risk mechanisms of ASD [152, 153].

### Summary

Multiple promising endophenotypes associated with key behavioral traits in ASD, including social, communication/language, cognitive, and sensorimotor behaviors, have been identified. Their analysis across multiple levels holds promise for identifying ETDs that may elucidate pathways of ASD inheritance. Analysis of ETDs is aided by focus on discrete, quantitative, and heritable traits that also are capable of being readily back-translated to model systems for interrogating cellular and molecular processes. The ETDs described above are not intended to be exhaustive, but instead serve as important targets for dense phenotyping family studies as has been done for separate psychiatric traits and clinical populations (e.g., [237–239]). Analyses of separate behaviors implicated in ASD (e.g., attention, sleep disturbances), and ETDs within the same individuals and across species will be imperative for mapping causal chains related to separate clinical traits in ASD.

## Bottom-up approaches for parsing etiological heterogeneity in ASD

The ETDs described above each were developed based on descriptions of behavioral traits associated with ASD. While we propose that “top-down” mapping of ETDs starting with behavioral traits will accelerate progress in understanding inherited pathways, it also is likely that significant advances will be generated using a “bottom-up” approach in which traits with simpler genetic structures serve as the starting point for connecting genes to behavior. This approach involves developing ETDs that leverage known genetic/molecular targets, blood-based, neurophysiological, or cellular traits to understand neurobiological processes contributing to select behavioral outcomes [240, 241]. Multiple candidates have been implicated based on their associations with autistic traits and ability to provide mechanistic insights, including increased extra-axial fluid [242–244], atypical melatonin [245], and immune system dysfunctions [246]. Studies are needed to determine the familiality of these traits and the extent to which they may offer power for more direct linkage with pathogenic variants.

Analysis of serotonergic (5HT) function represents an illustrative example of how an endophenotype-first approach may provide traction for gene discovery in ASD. Elevated whole-blood 5HT (WB5HT), or hyperserotonemia, represents perhaps the most replicated biomarker associated with ASD. It is evident in more than 25% of autistic individuals, tracks in unaffected family members [247, 248], and is highly heritable [249]. Despite WB5HT being a more molecular trait than the behaviors described above, mechanisms of hyperserotonemia remain complex and not well understood. It is possible that hyperserotonemia in ASD reflects atypicalities in synthesis within the intestines, abnormal uptake into platelet, or differences in 5HT release related to receptor expression or function [249–251]. One candidate mechanism that has been well studied is 5HT_2a_ receptor function in platelet aggregation. The vast majority (99%) of blood 5HT is stored in platelets [251], and 5HT_2a_ receptor enhances platelet aggregation [252] and is correlated with WB5HT levels [251, 253]. While findings regarding 5HT_2a_ receptors in platelets have not been consistent in ASD studies [254, 255], hyperserotonemic first-degree relatives of autistic children show lower densities of 5HT_2_ receptors in platelets in comparison to normoserotonemic relatives [251]. Further, PET and SPECT studies have indicated that individuals with ASD and parents in multiplex families show lower 5HT_2_ density and binding in cortex that may be negatively correlated with platelet 5HT [253]. While associations between 5HT_2a_ binding, WB5HT, and discrete behavioral traits in ASD have been largely inconsistent, these results suggest that WB5HT and 5HT_2a_ receptor density and function could serve as important candidate traits for taking an endophenotype-first approach to characterize genetic substructures in subsets of families, and is highly heritable [249]. Despite WB5HT being a more molecular trait than the behaviors described above, mechanisms of hyperserotonemia remain complex and not well understood. It is possible that hyperserotonemia in ASD reflects atypicalities in synthesis within the intestines, abnormal uptake into platelet, or differences in 5HT release related to receptor expression or function [249–251]. One candidate mechanism that has been well studied is 5HT_2a_ receptor function in platelet aggregation. The vast majority (~ 99%) of blood 5HT is stored in platelets [251], and 5HT_2a_ receptor enhances platelet aggregation [252] and is correlated with WB5HT levels [251, 253]. While findings regarding 5HT_2a_ receptors in platelets have not been consistent in ASD studies [254, 255], hyperserotonemic first-degree relatives of autistic children show lower densities of 5HT_2a_5HT_2_ receptors in platelets in comparison to normoserotonemic relatives [251]. Further, PET and SPECT studies have indicated that individuals with ASD and parents in multiplex families (i.e., families with more than one autistic child) show lower 5HT_2a_5HT_2_ density and binding in cortex that may be negatively correlated with platelet 5HT [253]. While associations between 5HT_2a_ binding, WB5HT, and discrete behavioral traits in ASD have been largely inconsistent, these results suggest that WB5HT and 5HT_2a_ receptor density and function could serve as important candidate traits for mapping ETDs to characterize genetic substructures of select traits in subsets of families.

Increased total brain volume is perhaps the most replicated finding from MRI studies of autistic individuals [256–258]. Brain enlargement also has been documented down to infancy [259, 260] with altered growth trajectories observed across the lifespan [256] involving multiple brain regions and subcortical structures and both gray and white matter [257, 260–263]. Importantly, head circumference, an index of total brain volume, has been found to be heritable [19]. Further, first-degree family members of individuals with ASD and macrocephaly show increased head circumference relative to family members of individuals with ASD who are not macrocephalic [240, 264], suggesting that co-segregation of autistic traits and macrocephaly may represent separate inherited polygenic backgrounds or biotypes. While increased head circumference has been a consistent finding, associations with clinical and behavioral traits have been inconsistent as studies have documented relationships with social-communication severity, restricted, repetitive behaviors, uneven cognitive development, and language outcomes [265–268], though these results have varied as a function of the age of individuals studied, their severity, clinical outcome measures, and brain regions studied [19, 269]. These “inconsistencies” are not surprising given the wide range of mechanisms that could underpin generalized brain overgrowth, and studies focused on select subregions or subcortical structures have found strong and somewhat consistent relationships with different clinical behaviors (e.g., [267, 268, 270]). Developmental variations also appear to strongly impact the nature and direction of these relationships [161].

Further, separate mechanisms may contribute to brain enlargement, and the extent to which they may be related to overlapping or distinct phenotypic patterns remains unclear. For example, germline mutations in PTEN, a tumor-suppressing gene, have been linked to conditions of tissue overgrowth in Cowden and Bannayan-Riley Ruvalcaba syndromes [271] and macrocephaly in some individuals with ASD. More specifically, 10–20% of autistic individuals with macrocephaly show PTEN mutations [271–273], and macrocephaly also is seen in non-autistic family members of these probands, but only selectively in family members who also have PTEN [272]. These findings are important in potentially understanding downstream molecular signaling mechanisms (e.g., mTOR) that may contribute to risk for autistic traits and macrocephaly, and for guiding imaging and neuropsychological studies to map ETDs useful for indexing etiological processes associated with autistic traits in populations with macrocephaly. Importantly, associations between macrocephaly, autistic traits, and PTEN mutations also inform powerful approaches for interrogating select trait pathways in model systems. For example, happloinsufficient mice with PTEN mutations also show brain overgrowth and some social and behavioral traits associated with ASD [274]. Similarly, selective deletion of PTEN in the hippocampus and layers III and V of cerebral cortex lead to macrocephaly in mice as well as atypical social behaviors, sensory hyperreactivity, and neuronal hypertrophy [275]. It remains unclear if molecular signaling and neurodevelopmental pathways impacted by PTEN mutations may overlap with those affecting some autistic individuals with macrocephaly but without known genetic causes. Studies of PTEN mutations provide a promising illustration of how endophenotype research may benefit from studies examining relationships between the many known genetic variants associated with autistic traits and candidate endophenotypes. Knowledge of the functional significance of known genetic risk variants also may be leveraged to generate new endophenotypes.

## Transdiagnostic considerations

While the focus of this review has been on ETDs associated with ASD, the endophenotype approach derives power from measuring traits that are continuously distributed in the population and agnostic to diagnostic status or categorical distinctions. This conceptual shift is critical in the context of repeated findings that the vast majority of variants associated with ASD also are associated with multiple separate psychiatric and neurodevelopmental disorders (e.g., [34, 276]). In support of this proposal, multiple studies have shown that copy number variants (CNVs) associated with ASD and other neuropsychiatric disorders (e.g., schizophrenia, ADHD) also converge on common patterns of functional and structural brain differences. Moreau et al. [277] identified signatures of functional connectivity differences associated with both 16p11.2 and 22q11.2 CNVs that both transcended clinical diagnosis (ASD, ADHD, schizophrenia) and also were seen in select cases without known associated variants. Studies of brain morphometry have shown similar overlap across different CNVs and subsets of individuals without known genetic causes for their diagnosis (i.e., idiopathic cases), suggesting that understanding of the neurodevelopmental processes affected by pathogenic CNVs may inform understanding of broader subgroups of neurodevelopmental traits [278]. Importantly, because these CNVs and associated clinical traits are present across diagnostic categories, they strongly suggest that transdiagnostic strategies will be crucial for understanding pathogenicity.

Historically, endophenotype criteria have stipulated that ETDs must be disorder-specific, but research in psychiatry has yielded strong evidence of phenotypic overlap at multiple levels of analysis. Population studies of non-clinical samples have indicated that, in many instances, self-endorsed clinical symptoms show similar levels of heterogeneity within diagnostic categories as between diagnostic categories [279]. Consistent with this hypothesis, the vast majority of (if not all) traits associated with ASD also are implicated in separate developmental conditions (e.g., atypical social gaze is seen in non-clinical populations as well as social phobia and schizophrenia; repetitive behaviors are seen in non-clinical populations, intellectual developmental disability and OCD). ETD discovery will be most powerful if focused on the full range of trait variation, including clinical and subclinical variation [280]. By measuring the full range of endophenotypic trait variation, we will have more power to identify associations with dimensional behavioral traits and neurodivergent trajectories without the constraints of categorical designations that do not appear to be directly linked to discrete biologies.

## Summary and conclusions

It is our position that understanding the complex pathogenic processes associated with ASD can be accelerated by shifting away from traditional case–control designs for gene discovery and instead prioritizing transdiagnostic studies that integrate dimensional endophenotypes for participant stratification. One method for leveraging candidate endophenotypes to understand mechanisms of ASD inheritance is developing ETDs, or associated endophenotypes that cut across functional units of analysis. Similar approaches (e.g., see above descriptions of the BSNIP studies) have proven powerful for identifying more biologically homogeneous subgroups of individuals, but have not yet been leveraged to understand traits associated with neurodevelopmental disorders including ASD. The quest for ETDs involved in ASD should focus on traits that are discrete, quantitative, familial, closer to gene action than behavioral traits, and translational. The dimensional and condition-agnostic nature of ETDs may accelerate gene discovery and advance understanding of heritable, polygenic processes contributing to neurodevelopmental impairments.

## Data Availability

Not applicable.

## References

[1] Neale BM, Kou Y, Liu L, Ma’Ayan A, Samocha KE, Sabo A (2012). Patterns and rates of exonic de novo mutations in autism spectrum disorders. Nature.

[2] Sanders SJ, Murtha MT, Gupta AR, Murdoch JD, Raubeson MJ, Willsey AJ (2012). De novo mutations revealed by whole-exome sequencing are strongly associated with autism. Nature.

[3] Persico AM, Van de Water J, Pardo CA. Autism: Where Genetics Meets the Immune System. Autism Res Treat. 2012;2012:486359. 10.1155/2012/486359 PMC3440848 22988503

[4] Gaugler T, Klei L, Sanders SJ, Bodea CA, Goldberg AP, Lee AB (2014). Most genetic risk for autism resides with common variation. Nat Genet.

[5] Toma C (2020). Genetic variation across phenotypic severity of autism. Trends Genet.

[6] Murphy D, Spooren W (2012). EU-AIMS: a boost to autism research. Nat Rev Drug Discov.

[7] Christensen ZP, Freedman EG, Foxe JJ. Autism is Associated with in vivo Changes in Gray Matter Neurite Architecture. bioRxiv. 2023:2023.03. 25.534208. 10.1002/aur.3239 39324563

[8] Loth E, Spooren W, Murphy DG (2014). New treatment targets for autism spectrum disorders: EU-AIMS. Lancet Psychiatry.

[9] Gui Y, Zhou X, Wang Z, Zhang Y, Wang Z, Zhou G (2022). Sex-specific genetic association between psychiatric disorders and cognition, behavior and brain imaging in children and adults. Transl Psychiatry.

[10] Kalsner L, Twachtman-Bassett J, Derynioski L, Chamberlain S. Yield of genetic testing including targeted gene panel in a clinical population of children with autism spectrum disorder (P1. 303). AAN Enterprises; 2018. 10.1002/mgg3.354 PMC5902398 29271092

[11] Tammimies K, Marshall CR, Walker S, Kaur G, Thiruvahindrapuram B, Lionel AC (2015). Molecular diagnostic yield of chromosomal microarray analysis and whole-exome sequencing in children with autism spectrum disorder. JAMA.

[12] Erdmann J, Großhennig A, Braund PS, König IR, Hengstenberg C, Hall AS (2009). New susceptibility locus for coronary artery disease on chromosome 3q22. 3. Nat Genet.

[13] Sing CF, Stengârd JH, Kardia SL (2003). Genes, environment, and cardiovascular disease. Arterioscler Thromb Vasc Biol.

[14] Comuzzie AG, Hixson JE, Almasy L, Mitchell BD, Mahaney MC, Dyer TD (1997). A major quantitative trait locus determining serum leptin levels and fat mass is located on human chromosome 2. Nat Genet.

[15] Consortium TG (2009). Six new loci associated with body mass index highlight a neuronal influence on body weight regulation. Nat Genet.

[16] Hsueh W-C, Mitchell BD, Aburomia R, Pollin T, Sakul H, Gelder Ehm M (2000). Diabetes in the Old Order Amish: characterization and heritability analysis of the Amish Family Diabetes Study. Diabetes Care.

[17] Demissie S, Dupuis J, Cupples L, Beck T, Kiel D, Karasik D (2007). Proximal hip geometry is linked to several chromosomal regions: genome-wide linkage results from the Framingham Osteoporosis Study. Bone.

[18] Mitchell BD, Kammerer CM, Schneider JL, Perez R, Bauer RL (2003). Genetic and environmental determinants of bone mineral density in Mexican Americans: results from the San Antonio Family Osteoporosis Study. Bone.

[19] Sacco R, Gabriele S, Persico AM (2015). Head circumference and brain size in autism spectrum disorder: a systematic review and meta-analysis. Psychiatry Res Neuroimaging.

[20] Tiede GM, Walton KM (2021). Social endophenotypes in autism spectrum disorder: a scoping review. Dev Psychopathol.

[21] Tamminga CA, Clementz BA, Pearlson G, Keshavan M, Gershon ES, Ivleva EI (2021). Biotyping in psychosis: using multiple computational approaches with one data set. Neuropsychopharmacology.

[22] Sanislow CA, Ferrante M, Pacheco J, Rudorfer MV, Morris SE (2019). Advancing translational research using NIMH research domain criteria and computational methods. Neuron.

[23] Hyman SE (2021). Psychiatric disorders: grounded in human biology but not natural kinds. Perspect Biol Med.

[24] John B, Lewis KR (1966). Chromosome variability and geographic distribution in insects: chromosome rather than gene variations provide the key to differences among populations. Science.

[25] Gottesman II, Shields J (1972). Schizophrenia and genetics. A twin study vantage point.

[26] Gottesman II, Gould TD (2003). The endophenotype concept in psychiatry: etymology and strategic intentions. Am J Psychiatry.

[27] Glahn DC, Curran JE, Winkler AM, Carless MA, Kent JW, Charlesworth JC (2012). High dimensional endophenotype ranking in the search for major depression risk genes. Biol Psychiatry.

[28] Gould TD, Gottesman II (2006). Psychiatric endophenotypes and the development of valid animal models. Genes Brain Behav.

[29] Gershon ES, Goldin L (1986). Clinical methods in psychiatric genetics: I. Robustness of genetic marker investigative strategies. Acta Psychiatr Scand.

[30] Elison JT (2020). Considering transient instantiators. Dev Psychopathol.

[31] Bryson SE, Zwaigenbaum L (2014). Autism observation scale for infants. Comprehensive guide to autism.

[32] Mous SE, Jiang A, Agrawal A, Constantino JN (2017). Attention and motor deficits index non-specific background liabilities that predict autism recurrence in siblings. J Neurodev Disord.

[33] Association AP (2013). Diagnostic and statistical manual of mental disorders (DSM-5 (R)).

[34] Carroll LS, Owen MJ (2009). Genetic overlap between autism, schizophrenia and bipolar disorder. Genome Med.

[35] Ma S-L, Chen LH, Lee C-C, Lai KY, Hung S-F, Tang C-P (2021). Genetic overlap between attention deficit/hyperactivity disorder and autism spectrum disorder in SHANK2 gene. Front Neurosci.

[36] González-Peñas J, Costas JC, García-Alcón A, Penzol MJ, Rodríguez J, Rodríguez-Fontenla C (2020). Psychiatric comorbidities in Asperger syndrome are related with polygenic overlap and differ from other Autism subtypes. Transl Psychiatry.

[37] Constantino JN, Todd RD (2003). Autistic traits in the general population: a twin study. Arch Gen Psychiatry.

[38] Lundström S, Chang Z, Råstam M, Gillberg C, Larsson H, Anckarsäter H (2012). Autism spectrum disorders and autisticlike traits: similar etiology in the extreme end and the normal variation. Arch Gen Psychiatry.

[39] Astle DE, Holmes J, Kievit R, Gathercole SE (2022). Annual research review: the transdiagnostic revolution in neurodevelopmental disorders. J Child Psychol Psychiatry.

[40] Ahmed AA, Vander Wyk BC (2013). Neural processing of intentional biological motion in unaffected siblings of children with autism spectrum disorder: an fMRI study. Brain Cogn.

[41] de Klerk CC, Gliga T, Charman T, Johnson MH, Team B (2014). Face engagement during infancy predicts later face recognition ability in younger siblings of children with autism. Dev Sci.

[42] Esposito G, del Carmen RM, Venuti P, Haltigan JD, Messinger DS (2014). Brief Report: Atypical expression of distress during the separation phase of the strange situation procedure in infant siblings at high risk for ASD. J Autism Dev Disord.

[43] Gliga T, Senju A, Pettinato M, Charman T, Johnson MH (2014). Spontaneous belief attribution in younger siblings of children on the autism spectrum. Dev Psychol.

[44] Lambert-Brown BL, McDonald NM, Mattson WI, Martin KB, Ibañez LV, Stone WL (2015). Positive emotional engagement and autism risk. Dev Psychol.

[45] Nichols CM, Ibañez LV, Foss-Feig JH, Stone WL (2014). Social smiling and its components in high-risk infant siblings without later ASD symptomatology. J Autism Dev Disord.

[46] Sheinkopf SJ, Iverson JM, Rinaldi ML, Lester BM (2012). Atypical cry acoustics in 6-month-old infants at risk for autism spectrum disorder. Autism Res.

[47] Webb SJ, Jones EJ, Merkle K, Namkung J, Toth K, Greenson J (2010). Toddlers with elevated autism symptoms show slowed habituation to faces. Child Neuropsychol.

[48] Seery A, Tager-Flusberg H, Nelson CA (2014). Event-related potentials to repeated speech in 9-month-old infants at risk for autism spectrum disorder. J Neurodev Disord.

[49] Kaiser MD, Hudac CM, Shultz S, Lee SM, Cheung C, Berken AM (2010). Neural signatures of autism. Proc Natl Acad Sci.

[50] Campbell SB, Moore EL, Northrup J, Brownell CA (2017). Developmental changes in empathic concern and self-understanding in toddlers at genetic risk for autism spectrum disorder. J Autism Dev Disord.

[51] Dalton KM, Nacewicz BM, Alexander AL, Davidson RJ (2007). Gaze-fixation, brain activation, and amygdala volume in unaffected siblings of individuals with autism. Biol Psychiatry.

[52] Gokcen S, Bora E, Erermis S, Kesikci H, Aydin C (2009). Theory of mind and verbal working memory deficits in parents of autistic children. Psychiatry Res.

[53] Holt R, Chura L, Lai M-C, Suckling J, Von Dem Hagen E, Calder A (2014). ‘Reading the mind in the eyes’: an fMRI study of adolescents with autism and their siblings. Psychol Med.

[54] Werling DM, Geschwind DH (2013). Sex differences in autism spectrum disorders. Curr Opin Neurol.

[55] Van Wijngaarden-Cremers PJ, van Eeten E, Groen WB, Van Deurzen PA, Oosterling IJ, Van der Gaag RJ (2014). Gender and age differences in the core triad of impairments in autism spectrum disorders: a systematic review and meta-analysis. J Autism Dev Disord.

[56] Floris DL, Lai M-C, Giavasis S, Oldehinkel M, Mennes M, Charman T (2021). Towards robust and replicable sex differences in the intrinsic brain function of autism. Mol Autism.

[57] Bloss CS, Courchesne E (2007). MRI neuroanatomy in young girls with autism: a preliminary study. J Am Acad Child Adolesc Psychiatry.

[58] Nordahl CW, Lange N, Li DD, Barnett LA, Lee A, Buonocore MH (2011). Brain enlargement is associated with regression in preschool-age boys with autism spectrum disorders. Proc Natl Acad Sci.

[59] Schaafsma SM, Pfaff DW (2014). Etiologies underlying sex differences in autism spectrum disorders. Front Neuroendocrinol.

[60] Bedford R, Jones EJ, Johnson MH, Pickles A, Charman T, Gliga T (2016). Sex differences in the association between infant markers and later autistic traits. Mol Autism.

[61] Burrows CA, Grzadzinski RL, Donovan K, Stallworthy IC, Rutsohn J, John TS (2022). A data-driven approach in an unbiased sample reveals equivalent sex ratio of autism spectrum disorder–associated impairment in early childhood. Biol Psychiatry.

[62] Messinger DS, Young GS, Webb SJ, Ozonoff S, Bryson SE, Carter A (2015). Early sex differences are not autism-specific: a baby siblings research consortium (BSRC) study. Mol Autism.

[63] Williams J, Blangero J (2004). Power of variance component linkage analysis—II. Discrete traits. Ann Hum Genet.

[64] Iacono WG, Malone SM, Vrieze SI (2017). Endophenotype best practices. Int J Psychophysiol.

[65] Glahn DC, Williams JT, McKay DR, Knowles EE, Sprooten E, Mathias SR (2015). Discovering schizophrenia endophenotypes in randomly ascertained pedigrees. Biol Psychiatry.

[66] Kaiser T, Feng G (2015). Modeling psychiatric disorders for developing effective treatments. Nat Med.

[67] Schmitt LM, Bojanek E, White SP, Ragozzino ME, Cook EH, Sweeney JA (2019). Familiality of behavioral flexibility and response inhibition deficits in autism spectrum disorder (ASD). Mol Autism.

[68] Nayar K, Sealock JM, Maltman N, Bush L, Cook EH, Davis LK, Losh M. Elevated polygenic burden for autism spectrum disorder is associated with the broad autism phenotype in mothers of individuals with autism spectrum disorder. Biol Psychiatry. 2021;89(5):476–85. 10.1016/j.biopsych.2020.08.029 PMC7901138 33229037

[69] Yoo HJ, Yang SY, Cho IH, Park M, Kim SA (2014). Polymorphisms of BDNF gene and autism spectrum disorders: family based association study with korean trios. Psychiatry Investig.

[70] Ozonoff S, Young GS, Carter A, Messinger D, Yirmiya N, Zwaigenbaum L (2011). Recurrence risk for autism spectrum disorders: a Baby Siblings Research Consortium study. Pediatrics.

[71] Sandin S, Lichtenstein P, Kuja-Halkola R, Larsson H, Hultman CM, Reichenberg A (2014). The familial risk of autism. JAMA.

[72] Christensen DL, Braun KVN, Baio J, Bilder D, Charles J, Constantino JN (2018). Prevalence and characteristics of autism spectrum disorder among children aged 8 years—autism and developmental disabilities monitoring network, 11 sites, United States, 2012. MMWR Surveill Summ.

[73] Zwaigenbaum L, Bryson S, Rogers T, Roberts W, Brian J, Szatmari P (2005). Behavioral manifestations of autism in the first year of life. Int J Dev Neurosci.

[74] Szatmari P, Chawarska K, Dawson G, Georgiades S, Landa R, Lord C (2016). Prospective longitudinal studies of infant siblings of children with autism: lessons learned and future directions. J Am Acad Child Adolesc Psychiatry.

[75] Livingston LA, Happé F (2017). Conceptualising compensation in neurodevelopmental disorders: reflections from autism spectrum disorder. Neurosci Biobehav Rev.

[76] Johnson MH, Jones EJ, Gliga T (2015). Brain adaptation and alternative developmental trajectories. Dev Psychopathol.

[77] Ivleva EI, Clementz BA, Dutcher AM, Arnold SJ, Jeon-Slaughter H, Aslan S (2017). Brain structure biomarkers in the psychosis biotypes: findings from the bipolar-schizophrenia network for intermediate phenotypes. Biol Psychiatry.

[78] Clementz BA, Trotti RL, Pearlson GD, Keshavan MS, Gershon ES, Keedy SK (2020). Testing psychosis phenotypes from bipolar–schizophrenia network for intermediate phenotypes for clinical application: biotype characteristics and targets. Biol Psychiatry Cogn Neurosci Neuroimaging.

[79] Constantino JN, Kennon-McGill S, Weichselbaum C, Marrus N, Haider A, Glowinski AL (2017). Infant viewing of social scenes is under genetic control and is atypical in autism. Nature.

[80] Kennedy DP, D’Onofrio BM, Quinn PD, Bölte S, Lichtenstein P, Falck-Ytter T (2017). Genetic influence on eye movements to complex scenes at short timescales. Curr Biol.

[81] Siqueiros Sanchez M, Pettersson E, Kennedy DP, Bölte S, Lichtenstein P, D’Onofrio BM (2020). Visual disengagement: genetic architecture and relation to autistic traits in the general population. J Autism Dev Disord.

[82] Siqueiros Sanchez M, Falck-Ytter T, Kennedy DP, Bölte S, Lichtenstein P, D’Onofrio BM (2020). Volitional eye movement control and ADHD traits: a twin study. J Child Psychol Psychiatry.

[83] Constantino JN (2014). Recurrence rates in autism spectrum disorders. JAMA.

[84] Hallmayer J, Cleveland S, Torres A, Phillips J, Cohen B, Torigoe T (2011). Genetic heritability and shared environmental factors among twin pairs with autism. Arch Gen Psychiatry.

[85] Ronald A, Hoekstra RA (2011). Autism spectrum disorders and autistic traits: a decade of new twin studies. Am J Med Genet B Neuropsychiatr Genet.

[86] Folstein S, Rutter M (1977). Genetic influences and infantile autism. Nature.

[87] Tick B, Bolton P, Happé F, Rutter M, Rijsdijk F (2016). Heritability of autism spectrum disorders: a meta-analysis of twin studies. J Child Psychol Psychiatry.

[88] Colvert E, Tick B, McEwen F, Stewart C, Curran SR, Woodhouse E (2015). Heritability of autism spectrum disorder in a UK population-based twin sample. JAMA Psychiatry.

[89] Sandin S, Lichtenstein P, Kuja-Halkola R, Hultman C, Larsson H, Reichenberg A (2017). The heritability of autism spectrum disorder. JAMA.

[90] Castelbaum L, Sylvester CM, Zhang Y, Yu Q, Constantino JN (2020). On the nature of monozygotic twin concordance and discordance for autistic trait severity: a quantitative analysis. Behav Genet.

[91] Lenzenweger MF (2013). Endophenotype, intermediate phenotype, biomarker: definitions, concept comparisons, clarifications. Depress Anxiety.

[92] Lenzenweger MF (2013). Thinking clearly about the endophenotype–intermediate phenotype–biomarker distinctions in developmental psychopathology research. Dev Psychopathol.

[93] Almasy L (2003). Quantitative risk factors as indices of alcoholism susceptibility. Ann Med.

[94] Piven J, Palmer P, Landa R, Santangelo S, Jacobi D, Childress D (1997). Personality and language characteristics in parents from multiple-incidence autism families. Am J Med Genet.

[95] Bolton P, Macdonald H, Pickles A, Rios PA, Goode S, Crowson M (1994). A case-control family history study of autism. J Child Psychol Psychiatry.

[96] Szatmari P, MacLean JE, Jones MB, Bryson SE, Zwaigenbaum L, Bartolucci G (2000). The familial aggregation of the lesser variant in biological and nonbiological relatives of PDD probands: a family history study. J Child Psychol Psychiatry Allied Discip.

[97] Losh M, Childress D, Lam K, Piven J (2008). Defining key features of the broad autism phenotype: a comparison across parents of multiple-and single-incidence autism families. Am J Med Genet B Neuropsychiatr Genet.

[98] Constantino JN, Zhang Y, Frazier T, Abbacchi AM, Law P (2010). Sibling recurrence and the genetic epidemiology of autism. Am J Psychiatry.

[99] Piven J, Wzorek M, Landa R, Lainhart J, Bolton P, Chase G (1994). Personality characteristics of the parents of autistic individuals. Psychol Med.

[100] Lyall K, Constantino JN, Weisskopf MG, Roberts AL, Ascherio A, Santangelo SL (2014). Parental social responsiveness and risk of autism spectrum disorder in offspring. JAMA Psychiatry.

[101] Frazier TW, Strauss M, Klingemier EW, Zetzer EE, Hardan AY, Eng C (2017). A meta-analysis of gaze differences to social and nonsocial information between individuals with and without autism. J Am Acad Child Adolesc Psychiatry.

[102] Jones W, Klin A (2013). Attention to eyes is present but in decline in 2–6-month-old infants later diagnosed with autism. Nature.

[103] Pelphrey KA, Sasson NJ, Reznick JS, Paul G, Goldman BD, Piven J (2002). Visual scanning of faces in autism. J Autism Dev Disord.

[104] Dalton KM, Nacewicz BM, Johnstone T, Schaefer HS, Gernsbacher MA, Goldsmith HH (2005). Gaze fixation and the neural circuitry of face processing in autism. Nat Neurosci.

[105] Adolphs R, Spezio ML, Parlier M, Piven J (2008). Distinct face-processing strategies in parents of autistic children. Curr Biol.

[106] Nayar K, Shic F, Winston M, Losh M (2022). A constellation of eye-tracking measures reveals social attention differences in ASD and the broad autism phenotype. Mol Autism.

[107] Asghari SZ, Farashi S, Bashirian S, Jenabi E (2021). Distinctive prosodic features of people with autism spectrum disorder: a systematic review and meta-analysis study. Sci Rep.

[108] Patel SP, Nayar K, Martin GE, Franich K, Crawford S, Diehl JJ (2020). An acoustic characterization of prosodic differences in autism spectrum disorder and first-degree relatives. J Autism Dev Disord.

[109] Esposito G, Venuti P (2010). Developmental changes in the fundamental frequency (f0) of infants’ cries: a study of children with Autism Spectrum Disorder. Early Child Dev Care.

[110] D’Cruz A-M, Ragozzino ME, Mosconi MW, Shrestha S, Cook EH, Sweeney JA (2013). Reduced behavioral flexibility in autism spectrum disorders. Neuropsychology.

[111] Mosconi M, Kay M, D’cruz A-M, Seidenfeld A, Guter S, Stanford L (2009). Impaired inhibitory control is associated with higher-order repetitive behaviors in autism spectrum disorders. Psychol Med.

[112] Schmitt LM, White SP, Cook EH, Sweeney JA, Mosconi MW (2018). Cognitive mechanisms of inhibitory control deficits in autism spectrum disorder. J Child Psychol Psychiatry.

[113] Hughes C, Leboyer M, Bouvard M (1997). Executive function in parents of children with autism. Psychol Med.

[114] Hughes C, Plumet MH, Leboyer M (1999). Towards a cognitive phenotype for autism: increased prevalence of executive dysfunction and superior spatial span amongst siblings of children with autism. J Child Psychol Psychiatry.

[115] Miller HL, Ragozzino ME, Cook EH, Sweeney JA, Mosconi MW (2015). Cognitive set shifting deficits and their relationship to repetitive behaviors in autism spectrum disorder. J Autism Dev Disord.

[116] Wang Z, Magnon GC, White SP, Greene RK, Vaillancourt DE, Mosconi MW (2015). Individuals with autism spectrum disorder show abnormalities during initial and subsequent phases of precision gripping. J Neurophysiol.

[117] Sacrey L-AR, Zwaigenbaum L, Bryson S, Brian J, Smith IM (2018). The reach-to-grasp movement in infants later diagnosed with autism spectrum disorder: a high-risk sibling cohort study. J Neurodev Disord.

[118] Leezenbaum NB, Iverson JM (2019). Trajectories of posture development in infants with and without familial risk for autism spectrum disorder. J Autism Dev Disord.

[119] Mosconi MW, Kay M, D’Cruz A-M, Guter S, Kapur K, Macmillan C (2010). Neurobehavioral abnormalities in first-degree relatives of individuals with autism. Arch Gen Psychiatry.

[120] Kang E, Keifer CM, Levy EJ, Foss-Feig JH, McPartland JC, Lerner MD (2018). Atypicality of the N170 event-related potential in autism spectrum disorder: a meta-analysis. Biol Psychiatry Cogn Neurosci Neuroimaging.

[121] McPartland J, Dawson G, Webb SJ, Panagiotides H, Carver LJ (2004). Event-related brain potentials reveal anomalies in temporal processing of faces in autism spectrum disorder. J Child Psychol Psychiatry.

[122] Sysoeva OV, Constantino JN, Anokhin AP (2018). Event-related potential (ERP) correlates of face processing in verbal children with autism spectrum disorders (ASD) and their first-degree relatives: a family study. Mol Autism.

[123] Dawson G, Webb SJ, Wijsman E, Schellenberg G, Estes A, Munson J (2005). Neurocognitive and electrophysiological evidence of altered face processing in parents of children with autism: implications for a model of abnormal development of social brain circuitry in autism. Dev Psychopathol.

[124] Shephard E, Milosavljevic B, Mason L, Elsabbagh M, Tye C, Gliga T (2020). Neural and behavioural indices of face processing in siblings of children with autism spectrum disorder (ASD): a longitudinal study from infancy to mid-childhood. Cortex.

[125] Spencer MD, Holt RJ, Chura LR, Suckling J, Calder AJ, Bullmore ET (2011). A novel functional brain imaging endophenotype of autism: the neural response to facial expression of emotion. Transl Psychiatry.

[126] Yucel G, Belger A, Bizzell J, Parlier M, Adolphs R, Piven J (2015). Abnormal neural activation to faces in the parents of children with autism. Cereb Cortex.

[127] Otto-Meyer S, Krizman J, White-Schwoch T, Kraus N (2018). Children with autism spectrum disorder have unstable neural responses to sound. Exp Brain Res.

[128] Patel SP, Kim JH, Larson CR, Losh M (2019). Mechanisms of voice control related to prosody in autism spectrum disorder and first-degree relatives. Autism Res.

[129] D’Cruz A-M. Event-related functional neuroimaging of reversal learning in autism spectrum disorders. University of Illinois at Chicago; 2013.

[130] Agam Y, Joseph RM, Barton JJ, Manoach DS (2010). Reduced cognitive control of response inhibition by the anterior cingulate cortex in autism spectrum disorders. Neuroimage.

[131] Thakkar KN, Polli FE, Joseph RM, Tuch DS, Hadjikhani N, Barton JJ (2008). Response monitoring, repetitive behaviour and anterior cingulate abnormalities in autism spectrum disorders (ASD). Brain.

[132] Padmanabhan A, Garver K, O’Hearn K, Nawarawong N, Liu R, Minshew N (2015). Developmental changes in brain function underlying inhibitory control in autism spectrum disorders. Autism Res.

[133] Unruh KE, Martin LE, Magnon G, Vaillancourt DE, Sweeney JA, Mosconi MW (2019). Cortical and subcortical alterations associated with precision visuomotor behavior in individuals with autism spectrum disorder. J Neurophysiol.

[134] Lepping RJ, McKinney WS, Magnon GC, Keedy SK, Wang Z, Coombes SA (2022). Visuomotor brain network activation and functional connectivity among individuals with autism spectrum disorder. Hum Brain Mapp.

[135] Floris DL, Barber AD, Nebel MB, Martinelli M, Lai M-C, Crocetti D (2016). Atypical lateralization of motor circuit functional connectivity in children with autism is associated with motor deficits. Mol Autism.

[136] Takagi M, Zee DS, Tamargo RJ (1998). Effects of lesions of the oculomotor vermis on eye movements in primate: saccades. J Neurophysiol.

[137] Mosconi MW, Luna B, Kay-Stacey M, Nowinski CV, Rubin LH, Scudder C (2013). Saccade adaptation abnormalities implicate dysfunction of cerebellar-dependent learning mechanisms in autism spectrum disorders (ASD). PLoS One.

[138] Chen Y, Yu J, Niu Y, Qin D, Liu H, Li G (2017). Modeling Rett syndrome using TALEN-edited MECP2 mutant cynomolgus monkeys. Cell.

[139] Machado CJ, Whitaker AM, Smith SE, Patterson PH, Bauman MD (2015). Maternal immune activation in nonhuman primates alters social attention in juvenile offspring. Biol Psychiatry.

[140] Crawley JN (2007). Mouse behavioral assays relevant to the symptoms of autism. Brain Pathol.

[141] Moy SS, Nadler JJ, Young NB, Nonneman RJ, Grossman AW, Murphy DL (2009). Social approach in genetically engineered mouse lines relevant to autism. Genes Brain Behav.

[142] Silverman J, Pride M, Hayes J, Puhger K, Butler-Struben H, Baker S (2015). GABAB receptor agonist R-baclofen reverses social deficits and reduces repetitive behavior in two mouse models of autism. Neuropsychopharmacology.

[143] Stoppel LJ, Kazdoba TM, Schaffler MD, Preza AR, Heynen A, Crawley JN (2018). R-baclofen reverses cognitive deficits and improves social interactions in two lines of 16p11. 2 deletion mice. Neuropsychopharmacology.

[144] Caruso A, Ricceri L, Scattoni ML (2020). Ultrasonic vocalizations as a fundamental tool for early and adult behavioral phenotyping of Autism Spectrum Disorder rodent models. Neurosci Biobehav Rev.

[145] Woehr M (2014). Ultrasonic vocalizations in Shank mouse models for autism spectrum disorders: detailed spectrographic analyses and developmental profiles. Neurosci Biobehav Rev.

[146] Amodeo DA, Jones JH, Sweeney JA, Ragozzino ME (2012). Differences in BTBR T+ tf/J and C57BL/6J mice on probabilistic reversal learning and stereotyped behaviors. Behav Brain Res.

[147] Arzuaga AL, Edmison DD, Mroczek J, Larson J, Ragozzino ME (2023). Prenatal stress and fluoxetine exposure in mice differentially affect repetitive behaviors and synaptic plasticity in adult male and female offspring. Behav Brain Res.

[148] Amodeo DA, Rivera E, Cook E, Sweeney JA, Ragozzino ME (2017). 5HT2A receptor blockade in dorsomedial striatum reduces repetitive behaviors in BTBR mice. Genes Brain Behav.

[149] Amodeo DA, Cuevas L, Dunn JT, Sweeney JA, Ragozzino ME (2018). The adenosine A2A receptor agonist, CGS 21680, attenuates a probabilistic reversal learning deficit and elevated grooming behavior in BTBR mice. Autism Res.

[150] Frankland P, Wang Y, Rosner B, Shimizu T, Balleine B, Dykens E (2004). Sensorimotor gating abnormalities in young males with fragile X syndrome and Fmr1-knockout mice. Mol Psychiatry.

[151] Al Sagheer T, Haida O, Balbous A, Francheteau M, Matas E, Fernagut P-O (2018). Motor impairments correlate with social deficits and restricted neuronal loss in an environmental model of autism. Int J Neuropsychopharmacol.

[152] Tsai PT, Hull C, Chu Y, Greene-Colozzi E, Sadowski AR, Leech JM (2012). Autistic-like behaviour and cerebellar dysfunction in Purkinje cell Tsc1 mutant mice. Nature.

[153] Tsai PT, Rudolph S, Guo C, Ellegood J, Gibson JM, Schaeffer SM (2018). Sensitive periods for cerebellar-mediated autistic-like behaviors. Cell Rep.

[154] Kanner L (1943). Autistic disturbances of affective contact. Nerv Child.

[155] Klin A, Jones W, Schultz R, Volkmar F, Cohen D (2002). Defining and quantifying the social phenotype in autism. Am J Psychiatry.

[156] Bentin S, Allison T, Puce A, Perez E, McCarthy G (1996). Electrophysiological studies of face perception in humans. J Cogn Neurosci.

[157] Anokhin AP, Golosheykin S, Heath AC (2010). Heritability of individual differences in cortical processing of facial affect. Behav Genet.

[158] Eimer M (2000). The face-specific N170 component reflects late stages in the structural encoding of faces. NeuroReport.

[159] Rossion B, Joyce CA, Cottrell GW, Tarr MJ (2003). Early lateralization and orientation tuning for face, word, and object processing in the visual cortex. Neuroimage.

[160] Webb SJ, Naples AJ, Levin AR, Hellemann G, Borland H, Benton J (2023). The autism biomarkers consortium for clinical trials: initial evaluation of a battery of candidate EEG biomarkers. Am J Psychiatry.

[161] Nacewicz BM, Dalton KM, Johnstone T, Long MT, McAuliff EM, Oakes TR (2006). Amygdala volume and nonverbal social impairment in adolescent and adult males with autism. Arch Gen Psychiatry.

[162] Kleinhans NM, Richards T, Sterling L, Stegbauer KC, Mahurin R, Johnson LC (2008). Abnormal functional connectivity in autism spectrum disorders during face processing. Brain.

[163] Sato W, Uono S (2019). The atypical social brain network in autism: advances in structural and functional MRI studies. Curr Opin Neurol.

[164] Zilbovicius M, Meresse I, Chabane N, Brunelle F, Samson Y, Boddaert N (2006). Autism, the superior temporal sulcus and social perception. Trends Neurosci.

[165] Watson KK, Platt ML (2012). Of mice and monkeys: using non-human primate models to bridge mouse-and human-based investigations of autism spectrum disorders. J Neurodev Disord.

[166] Mosher CP, Zimmerman PE, Gothard KM (2014). Neurons in the monkey amygdala detect eye contact during naturalistic social interactions. Curr Biol.

[167] Silverman JL, Yang M, Lord C, Crawley JN (2010). Behavioural phenotyping assays for mouse models of autism. Nat Rev Neurosci.

[168] Tager-Flusberg H, Caronna E (2007). Language disorders: autism and other pervasive developmental disorders. Pediatr Clin North Am.

[169] Lahvis GP, Alleva E, Scattoni ML (2011). Translating mouse vocalizations: prosody and frequency modulation 1. Genes Brain Behav.

[170] Lau JC, To CK, Kwan JS, Kang X, Losh M, Wong PC (2021). Lifelong tone language experience does not eliminate deficits in neural encoding of pitch in autism spectrum disorder. J Autism Dev Disord.

[171] Nayar K, Gordon PC, Martin GE, Hogan AL, La Valle C, McKinney W (2018). Links between looking and speaking in autism and first-degree relatives: insights into the expression of genetic liability to autism. Mol Autism.

[172] Nayar K, Sealock JM, Maltman N, Bush L, Cook EH, Davis LK (2021). Elevated polygenic burden for autism spectrum disorder is associated with the broad autism phenotype in mothers of individuals with autism spectrum disorder. Biol Psychiatry.

[173] Bidelman GM (2018). Subcortical sources dominate the neuroelectric auditory frequency-following response to speech. Neuroimage.

[174] Chandrasekaran B, Kraus N (2010). The scalp-recorded brainstem response to speech: neural origins and plasticity. Psychophysiology.

[175] Kraus N, Anderson S, White-Schwoch T. The frequency-following response: a window into human communication. Springer; 2017.

[176] Condouris K, Meyer E, Tager-Flusberg H. The relationship between standardized measures of language and measures of spontaneous speech in children with autism. 2003. 10.1044/1058-0360(2003/080) PMC1201513 12971823

[177] Friedman NP, Miyake A, Young SE, DeFries JC, Corley RP, Hewitt JK (2008). Individual differences in executive functions are almost entirely genetic in origin. J Exp Psychol Gen.

[178] Lee T, Mosing MA, Henry JD, Trollor JN, Ames D, Martin NG (2012). Genetic influences on four measures of executive functions and their covariation with general cognitive ability: the Older Australian Twins Study. Behav Genet.

[179] Baddeley A. The central executive and its malfunctions. Working memory. 1986. pp. 224–53.

[180] Baddeley AD. The development of the concept of working memory: implications and contributions of neuropsychology. 1990.

[181] Demetriou EA, DeMayo MM, Guastella AJ (2019). Executive function in autism spectrum disorder: history, theoretical models, empirical findings, and potential as an endophenotype. Front Psychiatry.

[182] Smith CJ, Lang CM, Kryzak L, Reichenberg A, Hollander E, Silverman JM (2009). Familial associations of intense preoccupations, an empirical factor of the restricted, repetitive behaviors and interests domain of autism. J Child Psychol Psychiatry.

[183] Szatmari P, Georgiades S, Bryson S, Zwaigenbaum L, Roberts W, Mahoney W (2006). Investigating the structure of the restricted, repetitive behaviours and interests domain of autism. J Child Psychol Psychiatry.

[184] Van Eylen L, Boets B, Cosemans N, Peeters H, Steyaert J, Wagemans J (2017). Executive functioning and local-global visual processing: candidate endophenotypes for autism spectrum disorder?. J Child Psychol Psychiatry.

[185] Ozonoff S, Rogers SJ, Farnham JM, Pennington BF (1993). Can standard measures identify subclinical markers of autism?. J Autism Dev Disord.

[186] Szatmari P, Jones MB, Tuff L, Bartolucci G, Fisman S, Mahoney W (1993). Lack of cognitive impairment in first-degree relatives of children with pervasive developmental disorders. J Am Acad Child Adolesc Psychiatry.

[187] Ghahremani DG, Monterosso J, Jentsch JD, Bilder RM, Poldrack RA (2010). Neural components underlying behavioral flexibility in human reversal learning. Cereb Cortex.

[188] Kok A, Ramautar JR, De Ruiter MB, Band GP, Ridderinkhof KR (2004). ERP components associated with successful and unsuccessful stopping in a stop-signal task. Psychophysiology.

[189] D’Cruz A-M, Ragozzino ME, Mosconi MW, Pavuluri MN, Sweeney JA (2011). Human reversal learning under conditions of certain versus uncertain outcomes. Neuroimage.

[190] Dichter GS, Radonovich KJ, Turner-Brown LM, Lam KS, Holtzclaw TN, Bodfish JW (2010). Performance of children with autism spectrum disorders on the dimension-change card sort task. J Autism Dev Disord.

[191] Kohls G, Schulte-Rüther M, Nehrkorn B, Müller K, Fink GR, Kamp-Becker I (2013). Reward system dysfunction in autism spectrum disorders. Soc Cogn Affect Neurosci.

[192] Mosconi MW, Sweeney JA (2015). Sensorimotor dysfunctions as primary features of autism spectrum disorders. Sci China Life Sci.

[193] Minshew NJ, Luna B, Sweeney JA (1999). Oculomotor evidence for neocortical systems but not cerebellar dysfunction in autism. Neurology.

[194] Barash S, Zhang M, editors. Switching of sensorimotor transformations: antisaccades and parietal cortex. In: Percept, decision, action: bridging the gaps. Novartis Foundation Symposium 270; 2006: Wiley Online Library. 16649708

[195] Everling S, Fischer B (1998). The antisaccade: a review of basic research and clinical studies. Neuropsychologia.

[196] Amador N, Schlag-Rey M, Schlag J (2004). Primate antisaccade. II. Supplementary eye field neuronal activity predicts correct performance. J Neurophysiol.

[197] Hardung S, Epple R, Jäckel Z, Eriksson D, Uran C, Senn V (2017). A functional gradient in the rodent prefrontal cortex supports behavioral inhibition. Curr Biol.

[198] Johnson BP, Lum JA, Rinehart NJ, Fielding J (2016). Ocular motor disturbances in autism spectrum disorders: systematic review and comprehensive meta-analysis. Neurosci Biobehav Rev.

[199] Lim YH, Partridge K, Girdler S, Morris SL (2017). Standing postural control in individuals with autism spectrum disorder: systematic review and meta-analysis. J Autism Dev Disord.

[200] Bruyneel E, Demurie E, Warreyn P, Roeyers H (2019). The mediating role of joint attention in the relationship between motor skills and receptive and expressive language in siblings at risk for autism spectrum disorder. Infant Behav Dev.

[201] Choi B, Leech KA, Tager-Flusberg H, Nelson CA (2018). Development of fine motor skills is associated with expressive language outcomes in infants at high and low risk for autism spectrum disorder. J Neurodev Disord.

[202] Flanagan JE, Landa R, Bhat A, Bauman M (2012). Head lag in infants at risk for autism: a preliminary study. Am J Occup Ther.

[203] Franchini M, Duku E, Armstrong V, Brian J, Bryson S, Garon N (2018). Variability in verbal and nonverbal communication in infants at risk for autism spectrum disorder: predictors and outcomes. J Autism Dev Disord.

[204] Garrido D, Petrova D, Watson LR, Garcia-Retamero R, Carballo G (2017). Language and motor skills in siblings of children with autism spectrum disorder: a meta-analytic review. Autism Res.

[205] Iverson JM, Shic F, Wall CA, Chawarska K, Curtin S, Estes A (2019). Early motor abilities in infants at heightened versus low risk for ASD: a baby siblings research consortium (BSRC) study. J Abnorm Psychol.

[206] LeBarton ES, Landa RJ (2019). Infant motor skill predicts later expressive language and autism spectrum disorder diagnosis. Infant Behav Dev.

[207] West KL (2019). Infant motor development in autism spectrum disorder: a synthesis and meta-analysis. Child Dev.

[208] Coll S-M, Foster NE, Meilleur A, Brambati SM, Hyde KL (2020). Sensorimotor skills in autism spectrum disorder: a meta-analysis. Res Autism Spect Dis.

[209] Mosconi MW, Mohanty S, Greene RK, Cook EH, Vaillancourt DE, Sweeney JA (2015). Feedforward and feedback motor control abnormalities implicate cerebellar dysfunctions in autism spectrum disorder. J Neurosci.

[210] Wang Z, Hallac RR, Conroy KC, White SP, Kane AA, Collinsworth AL (2016). Postural orientation and equilibrium processes associated with increased postural sway in autism spectrum disorder (ASD). J Neurodev Disord.

[211] Glazebrook C, Gonzalez D, Hansen S, Elliott D (2009). The role of vision for online control of manual aiming movements in persons with autism spectrum disorders. Autism.

[212] Stein BE, Wallace MT, Stanford TR (1999). Development of multisensory integration: transforming sensory input into motor output. Ment Retard Dev Disabil Res Rev.

[213] Stein J, Glickstein M (1992). Role of the cerebellum in visual guidance of movement. Physiol Rev.

[214] Glickstein M (2000). How are visual areas of the brain connected to motor areas for the sensory guidance of movement?. Trends Neurosci.

[215] Stanfield AC, McIntosh AM, Spencer MD, Philip R, Gaur S, Lawrie SM (2008). Towards a neuroanatomy of autism: a systematic review and meta-analysis of structural magnetic resonance imaging studies. Eur Psychiatry.

[216] Schumann CM, Nordahl CW (2011). Bridging the gap between MRI and postmortem research in autism. Brain Res.

[217] Yip J, Soghomonian J-J, Blatt GJ (2007). Decreased GAD67 mRNA levels in cerebellar Purkinje cells in autism: pathophysiological implications. Acta Neuropathol.

[218] Bauman M, Kemper TL (1985). Histoanatomic observations of the brain in early infantile autism. Neurology.

[219] Yip J, Soghomonian JJ, Blatt GJ (2008). Increased GAD67 mRNA expression in cerebellar interneurons in autism: implications for Purkinje cell dysfunction. J Neurosci Res.

[220] Missitzi J, Gentner R, Misitzi A, Geladas N, Politis P, Klissouras V (2013). Heritability of motor control and motor learning. Physiol Rep.

[221] Anderson KM, Ge T, Kong R, Patrick LM, Spreng RN, Sabuncu MR (2021). Heritability of individualized cortical network topography. Proc Natl Acad Sci.

[222] Li Z, Huang J, Xu T, Wang Y, Li K, Zeng YW (2018). Neural mechanism and heritability of complex motor sequence and audiovisual integration: a healthy twin study. Hum Brain Mapp.

[223] Mosconi M, Vaillancourt D, Sweeney J, editors. Cortico-cerebellar dysfunctions associated with visuomotor abnormalities in autism spectrum disorder vary according to the quality of visual feedback. In: Neuropsychopharmacology. Nature Publishing Group; 2014.

[224] Schmitt LM, Cook EH, Sweeney JA, Mosconi MW (2014). Saccadic eye movement abnormalities in autism spectrum disorder indicate dysfunctions in cerebellum and brainstem. Mol Autism.

[225] Takarae Y, Luna B, Minshew NJ, Sweeney JA (2008). Patterns of visual sensory and sensorimotor abnormalities in autism vary in relation to history of early language delay. J Int Neuropsychol Soc.

[226] Takarae Y, Minshew NJ, Luna B, Sweeney JA (2007). Atypical involvement of frontostriatal systems during sensorimotor control in autism. Psychiatry Res Neuroimaging.

[227] Patterson JW, Armstrong V, Duku E, Richard A, Franchini M, Brian J (2022). Early trajectories of motor skills in infant siblings of children with autism spectrum disorder. Autism Res.

[228] Leonard HC, Bedford R, Charman T, Elsabbagh M, Johnson MH, Hill EL (2014). Motor development in children at risk of autism: a follow-up study of infant siblings. Autism.

[229] Moruzzi S, Ogliari A, Ronald A, Happé F, Battaglia M (2011). The nature of covariation between autistic traits and clumsiness: a twin study in a general population sample. J Autism Dev Disord.

[230] Ozonoff S, Young GS, Belding A, Hill M, Hill A, Hutman T (2014). The broader autism phenotype in infancy: when does it emerge?. J Am Acad Child Adolesc Psychiatry.

[231] Caruso A, Gila L, Fulceri F, Salvitti T, Micai M, Baccinelli W (2020). Early motor development predicts clinical outcomes of siblings at high-risk for autism: insight from an innovative motion-tracking technology. Brain Sci.

[232] Leigh RJ, Zee DS. The neurology of eye movements: contemporary neurology. 2015.

[233] Takarae Y, Minshew NJ, Luna B, Krisky CM, Sweeney JA (2004). Pursuit eye movement deficits in autism. Brain.

[234] Dundas EM, Plaut DC, Behrmann M (2015). Variable left-hemisphere language and orthographic lateralization reduces right-hemisphere face lateralization. J Cogn Neurosci.

[235] Zang Y, De Schutter E (2019). Climbing fibers provide graded error signals in cerebellar learning. Front Syst Neurosci.

[236] Lijam N, Paylor R, McDonald MP, Crawley JN, Deng C-X, Herrup K (1997). Social interaction and sensorimotor gating abnormalities in mice lacking Dvl1. Cell.

[237] Tamminga CA, Pearlson G, Keshavan M, Sweeney J, Clementz B, Thaker G (2014). Bipolar and schizophrenia network for intermediate phenotypes: outcomes across the psychosis continuum. Schizophr Bull.

[238] Hill SK, Reilly JL, Keefe RS, Gold JM, Bishop JR, Gershon ES (2013). Neuropsychological impairments in schizophrenia and psychotic bipolar disorder: findings from the Bipolar-Schizophrenia Network on Intermediate Phenotypes (B-SNIP) study. Am J Psychiatry.

[239] Ivleva EI, Bidesi AS, Keshavan MS, Pearlson GD, Meda SA, Dodig D (2013). Gray matter volume as an intermediate phenotype for psychosis: bipolar-schizophrenia network on intermediate phenotypes (B-SNIP). Am J Psychiatry.

[240] Sacco R, Curatolo P, Manzi B, Militerni R, Bravaccio C, Frolli A (2010). Principal pathogenetic components and biological endophenotypes in autism spectrum disorders. Autism Res.

[241] Sacco R, Lenti C, Saccani M, Curatolo P, Manzi B, Bravaccio C (2012). Cluster analysis of autistic patients based on principal pathogenetic components. Autism Res.

[242] Shen MD, Nordahl CW, Li DD, Lee A, Angkustsiri K, Emerson RW (2018). Extra-axial cerebrospinal fluid in high-risk and normal-risk children with autism aged 2–4 years: a case-control study. Lancet Psychiatry.

[243] Shen MD, Kim SH, McKinstry RC, Gu H, Hazlett HC, Nordahl CW (2017). Increased extra-axial cerebrospinal fluid in high-risk infants who later develop autism. Biol Psychiatry.

[244] Shen MD, Nordahl CW, Young GS, Wootton-Gorges SL, Lee A, Liston SE (2013). Early brain enlargement and elevated extra-axial fluid in infants who develop autism spectrum disorder. Brain.

[245] Melke J, Goubran Botros H, Chaste P, Betancur C, Nygren G, Anckarsäter H (2008). Abnormal melatonin synthesis in autism spectrum disorders. Mol Psychiatry.

[246] Onore C, Careaga M, Ashwood P (2012). The role of immune dysfunction in the pathophysiology of autism. Brain Behav Immun.

[247] Mulder EJ, Anderson GM, Kema IP, De Bildt A, Van Lang ND, Den Boer JA (2004). Platelet serotonin levels in pervasive developmental disorders and mental retardation: diagnostic group differences, within-group distribution, and behavioral correlates. J Am Acad Child Adolesc Psychiatry.

[248] Stefano G, Sacco R, Persico AM (2014). Blood serotonin levels in autism spectrum disorder: a systematic review and meta-analysis. Eur Neuropsychopharmacol.

[249] Muller CL, Anacker AM, Veenstra-VanderWeele J (2016). The serotonin system in autism spectrum disorder: from biomarker to animal models. Neuroscience.

[250] Oblak A, Gibbs TT, Blatt GJ (2013). Reduced serotonin receptor subtypes in a limbic and a neocortical region in autism. Autism Res.

[251] Cook E, Arora RC, Anderson GM, Berry-Kravis EM, Yau S, Yeoh H (1993). Platelet serotonin studies in familial hyperserotonemia of autism. Life Sci.

[252] Oliver KH, Duvernay MT, Hamm HE, Carneiro AM (2016). Loss of serotonin transporter function alters ADP-mediated glycoprotein αIIbβ3 activation through dysregulation of the 5-HT2A receptor. J Biol Chem.

[253] Goldberg J, Anderson GM, Zwaigenbaum L, Hall GB, Nahmias C, Thompson A (2009). Cortical serotonin type-2 receptor density in parents of children with autism spectrum disorders. J Autism Dev Disord.

[254] McBride PA, Anderson GM, Hertzig ME, Sweeney JA, Kream J, Cohen DJ (1989). Serotonergic responsivity in male young adults with autistic disorder: results of a pilot study. Arch Gen Psychiatry.

[255] Perry BD, Cook EH, Leventhal BL, Wainwright MS, Freedman DX (1991). Platelet 5-HT2 serotonin receptor binding sites in autistic children and their first-degree relatives. Biol Psychiatry.

[256] Courchesne E, Campbell K, Solso S (2011). Brain growth across the life span in autism: age-specific changes in anatomical pathology. Brain Res.

[257] Redcay E, Courchesne E (2005). When is the brain enlarged in autism? A meta-analysis of all brain size reports. Biol Psychiatry.

[258] Piven J, Arndt S, Bailey J, Havercamp S, Andreasen NC, Palmer P (1995). An MRI study of brain size in autism. Am J Psychiatry.

[259] Hazlett HC, Gu H, Munsell BC, Kim SH, Styner M, Wolff JJ (2017). Early brain development in infants at high risk for autism spectrum disorder. Nature.

[260] Hazlett HC, Poe MD, Gerig G, Styner M, Chappell C, Smith RG (2011). Early brain overgrowth in autism associated with an increase in cortical surface area before age 2 years. Arch Gen Psychiatry.

[261] Wassink TH, Hazlett HC, Epping EA, Arndt S, Dager SR, Schellenberg GD (2007). Cerebral cortical gray matter overgrowth and functional variation of the serotonin transporter gene in autism. Arch Gen Psychiatry.

[262] Sparks B, Friedman S, Shaw D, Aylward EH, Echelard D, Artru A (2002). Brain structural abnormalities in young children with autism spectrum disorder. Neurology.

[263] Hazlett HC, Poe MD, Gerig G, Smith RG, Piven J (2006). Cortical gray and white brain tissue volume in adolescents and adults with autism. Biol Psychiatry.

[264] Sacco R, Militerni R, Frolli A, Bravaccio C, Gritti A, Elia M (2007). Clinical, morphological, and biochemical correlates of head circumference in autism. Biol Psychiatry.

[265] Deutsch CK, Joseph RM (2003). Brief report: cognitive correlates of enlarged head circumference in children with autism. J Autism Dev Disord.

[266] Munson J, Dawson G, Abbott R, Faja S, Webb SJ, Friedman SD (2006). Amygdalar volume and behavioral development in autism. Arch Gen Psychiatry.

[267] Schumann CM, Barnes CC, Lord C, Courchesne E (2009). Amygdala enlargement in toddlers with autism related to severity of social and communication impairments. Biol Psychiatry.

[268] Mosconi MW, Cody-Hazlett H, Poe MD, Gerig G, Gimpel-Smith R, Piven J (2009). Longitudinal study of amygdala volume and joint attention in 2-to 4-year-old children with autism. Arch Gen Psychiatry.

[269] Lainhart JE, Bigler ED, Bocian M, Coon H, Dinh E, Dawson G (2006). Head circumference and height in autism: a study by the Collaborative Program of Excellence in Autism. Am J Med Genet A.

[270] Wolff JJ, Hazlett HC, Lightbody AA, Reiss AL, Piven J (2013). Repetitive and self-injurious behaviors: associations with caudate volume in autism and fragile X syndrome. J Neurodev Disord.

[271] Varga EA, Pastore M, Prior T, Herman GE, McBride KL (2009). The prevalence of PTEN mutations in a clinical pediatric cohort with autism spectrum disorders, developmental delay, and macrocephaly. Genet Med.

[272] McBride KL, Varga EA, Pastore MT, Prior TW, Manickam K, Atkin JF (2010). Confirmation study of PTEN mutations among individuals with autism or developmental delays/mental retardation and macrocephaly. Autism Res.

[273] Klein S, Sharifi-Hannauer P, Martinez-Agosto JA (2013). Macrocephaly as a clinical indicator of genetic subtypes in autism. Autism Res.

[274] Clipperton-Allen AE, Page DT (2014). Pten haploinsufficient mice show broad brain overgrowth but selective impairments in autism-relevant behavioral tests. Hum Mol Genet.

[275] Kwon C-H, Luikart BW, Powell CM, Zhou J, Matheny SA, Zhang W (2006). Pten regulates neuronal arborization and social interaction in mice. Neuron.

[276] McCarthy SE, Gillis J, Kramer M, Lihm J, Yoon S, Berstein Y (2014). De novo mutations in schizophrenia implicate chromatin remodeling and support a genetic overlap with autism and intellectual disability. Mol Psychiatry.

[277] Moreau CA, Urchs SG, Kuldeep K, Orban P, Schramm C, Dumas G (2020). Mutations associated with neuropsychiatric conditions delineate functional brain connectivity dimensions contributing to autism and schizophrenia. Nat Commun.

[278] Modenato C, Martin-Brevet S, Moreau CA, Rodriguez-Herreros B, Kumar K, Draganski B (2021). Lessons learned from neuroimaging studies of copy number variants: a systematic review. Biol Psychiatry.

[279] Newson JJ, Pastukh V, Thiagarajan TC (2021). Poor separation of clinical symptom profiles by DSM-5 disorder criteria. Front Psych.

[280] Beauchaine TP, Constantino JN (2017). Redefining the endophenotype concept to accommodate transdiagnostic vulnerabilities and etiological complexity. Biomark Med.

