# Correction: Endophenotype trait domains for advancing gene discovery in autism spectrum disorder

**DOI:** 10.1186/s11689-024-09523-2

**Published:** 2024-02-28

**Authors:** Matthew W. Mosconi, Cassandra J. Stevens, Kathryn E. Unruh, Robin Shafer, Jed T. Elison

**Affiliations:** 1 Schiefelbusch Institute for Life Span Studies and Kansas Center for Autism Research and Training (K‑CART), University of Kansas, Lawrence, KS USA; 2 Clinical Child Psychology Program, University of Kansas, Lawrence, KS USA; 3 Institute of Child Development, University of Minnesota, Minneapolis, MN USA; 4 Department of Pediatrics, University of Minnesota, Minneapolis, MN USA

**Correction: J Neurodevelop Disord 15, 41 (2023)**

10.1186/s11689-023-09511-y

Following publication of the original article [1], the author reported that the wrong version of Fig. 1 which contains a PowerPoint background was uploaded.

Incorrect figure:

**Fig. 1:**
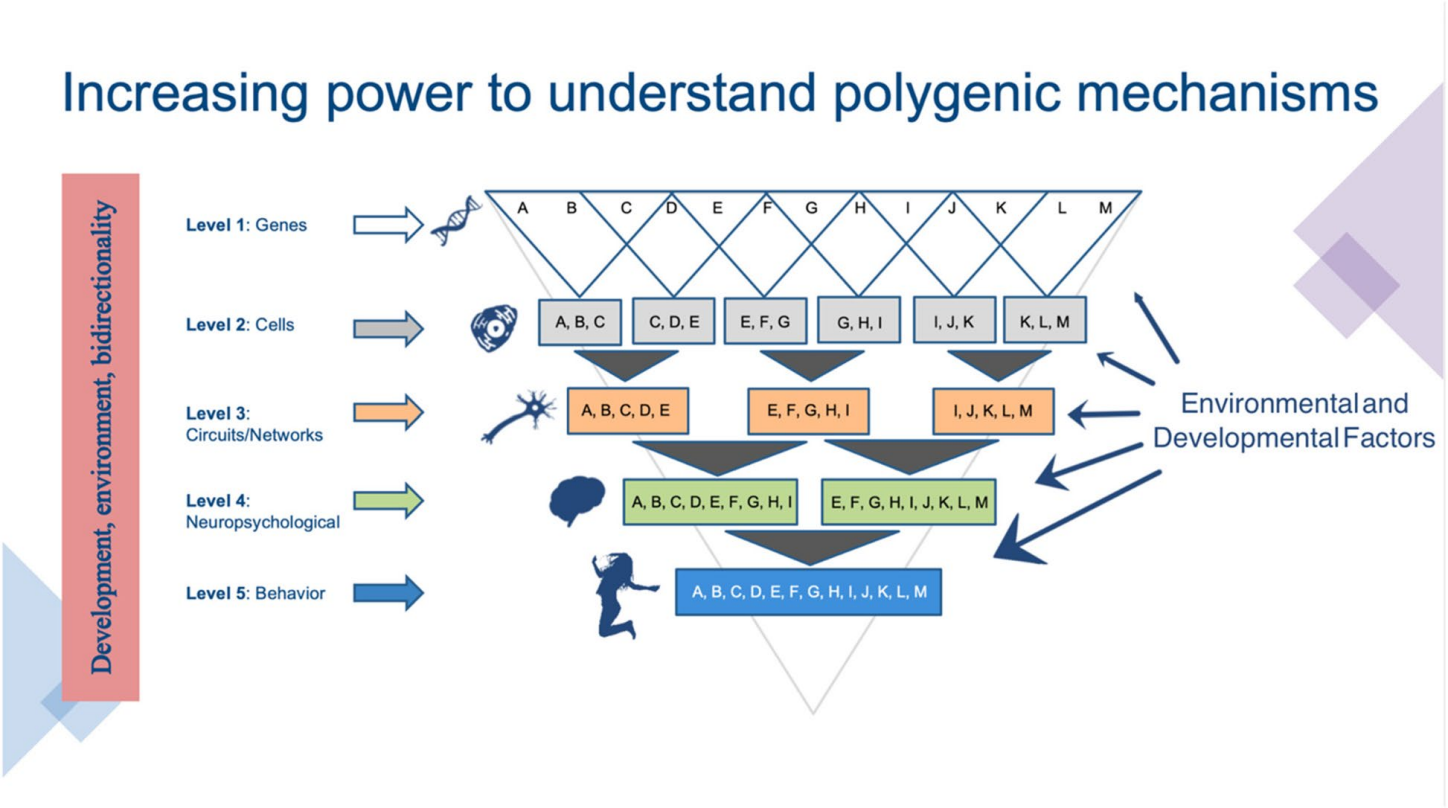
Levels of analysis for mapping etiological pathways associated with behavioral and clinical traits. This schematic shows different layers or functional units of analysis that can be evaluated to clarify linkages between genotype and clinical phenotype. Endophenotypic traits closer to the level of genotype are expected to be more closely associated with inherited variation given their relatively simpler genetic architecture compared to behavioral traits, as evidenced above by the reduced complexity (i.e., number of genes or letters) at the higher levels (e.g., cells, circuits/networks). Multiple levels of analysis are depicted, though separate intermediate levels are not included for ease of presentation (e.g., proteomic). Based on this model, analysis of traits closer to genotypes will provide greater sensitivity to inherited variations than assessments of behavior or complex clusters of clinical symptoms. Analysis of traits across multiple levels, or establishment of endophenotypic trait domains (ETDs), also is proposed to offer unique opportunities for understanding etiological pathways contributing to discrete traits associated with ASD. Important environmental and developmental factors also are proposed to modify trait associations across levels and over time

Correct figure:

**Fig. 1:**
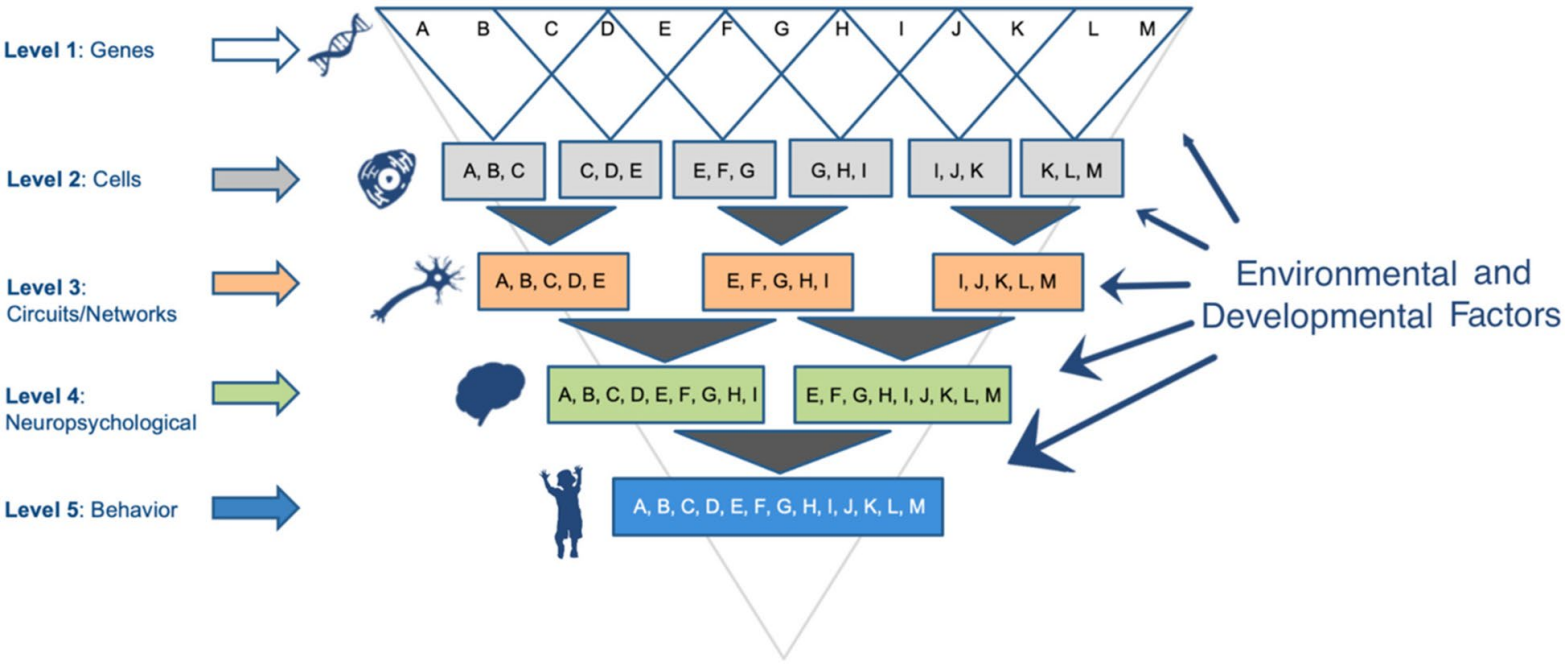
Levels of analysis for mapping etiological pathways associated with behavioral and clinical traits. This schematic shows different layers or functional units of analysis that can be evaluated to clarify linkages between genotype and clinical phenotype. Endophenotypic traits closer to the level of genotype are expected to be more closely associated with inherited variation given their relatively simpler genetic architecture compared to behavioral traits, as evidenced above by the reduced complexity (i.e., number of genes or letters) at the higher levels (e.g., cells, circuits/networks). Multiple levels of analysis are depicted, though separate intermediate levels are not included for ease of presentation (e.g., proteomic). Based on this model, analysis of traits closer to genotypes will provide greater sensitivity to inherited variations than assessments of behavior or complex clusters of clinical symptoms. Analysis of traits across multiple levels, or establishment of endophenotypic trait domains (ETDs), also is proposed to offer unique opportunities for understanding etiological pathways contributing to discrete traits associated with ASD. Important environmental and developmental factors also are proposed to modify trait associations across levels and over time

The original article has been corrected.
